# Supplementary figures and images for: Glioblastoma cells vampirize WNT from neurons and trigger a JNK/MMP signaling loop that enhances glioblastoma progression and neurodegeneration
Source: PLoS Biol. 2019 Dec 17;17(12):e3000545. doi: 10.1371/journal.pbio.3000545 (PMC6917273; doi:10.1371/journal.pbio.3000545)

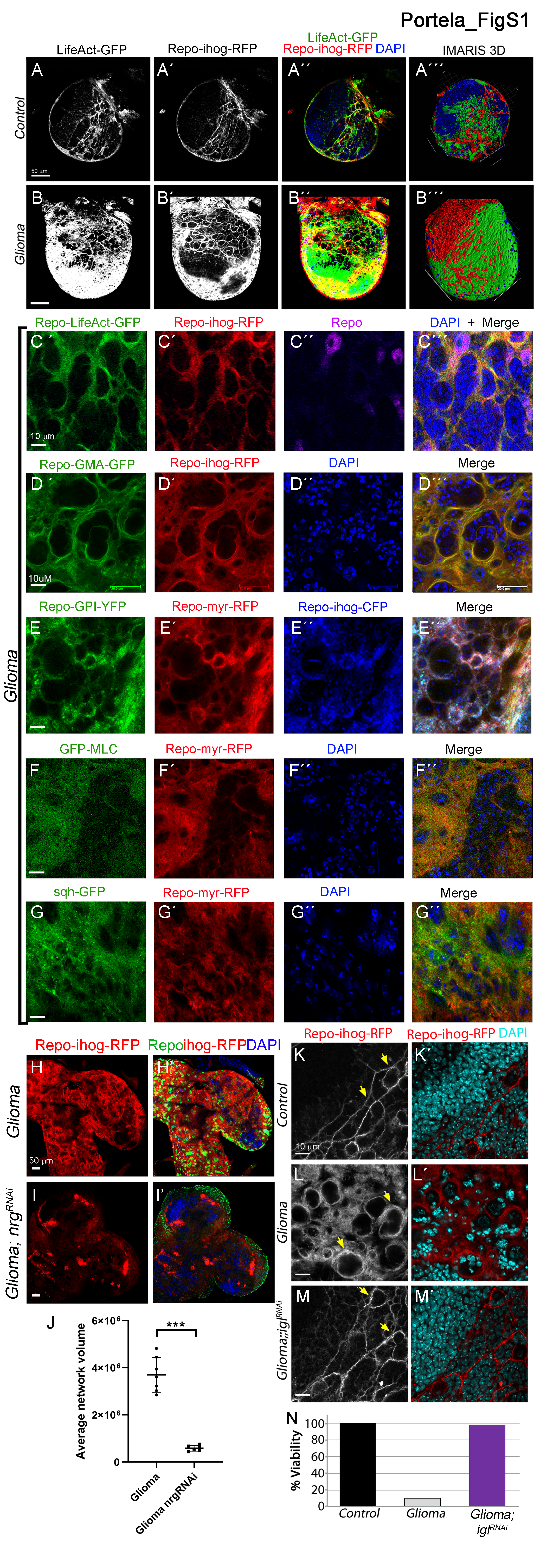

Supplement: S1 Fig — (A–B) Control and glioma brains from third instar larvae. Glia is labeled with UAS-Ihog-RFP (gray or red in the merge) driven by repo-Gal4 to visualize TMs in glial cells as part of an interconnecting network. The glial network is marked with lifeActin-GFP reporter (gray or green in the merge), and nuclei are marked with DAPI (blue). Imaris 3D reconstructions are shown in panels A‴–B‴). (C–G) Glial network is marked with myr-RFP/ihog-RFP (red) and several additional cytoneme markers: (C) lifeact-GFP reporter (green and glial nuclei are marked with Repo, magenta), (D) GMA-GFP (green), (E) GPI-YFP (green), (F) GFP-MLC (green), (G) sqh-GFP (green) in a glioma brain. (H–J) Down-regulation of neuroglian (nrg-RNAi) in glioma brains results in defective TMs (I, red) compared with glioma brains (H); glial nuclei are marked by Repo (green), and TM network volume is quantified in panel J. (K–N) Higher magnifications of control brains (K) showing the glial cytonemes (gray or red in merge) compared with the glioma brains in which the TMs overgrow and enwrap neuronal clusters (L). Upon igl/Gap43 down-regulation, the glial network does not overgrow or enwrap neuronal clusters (M) and shows a pattern and size similar to the control. Arrows indicate glial cytonemes/TMs. (N) A viability assay shows that the lethality induced by the glioma is fully rescued upon knockdown of Gap43/igl. Nuclei are marked with DAPI (blue). Scale bar size are indicated in this and all figures. The data underlying this figure can be found in S1 Data. Genotypes: (A) w; lifeActin-GFP; repo-Gal4, UAS-ihog-RFP/UAS-lacZ, (B-C) UAS-dEGFRλ, UAS-dp110CAAX; lifeActin-GFP; repo-Gal4, UAS-ihog-RFP, (D) UAS-dEGFRλ, UAS-dp110CAAX; UAS-GMA-GFP; repo-Gal4, UAS-ihog-RFP, (E) UAS-dEGFRλ, UAS-dp110CAAX; UAS-GPI-YFP/Gal80ts; repo-Gal4, UAS-myrRFP, (F) UAS-dEGFRλ, UAS-dp110CAAX; Gal80ts; repo-Gal4, UAS-myrRFP/ UAS-GFP-sls(MLC), (G) UAS-dEGFRλ, UAS-dp110CAAX; Gal80ts; repo-Gal4, UAS-myrRFP/ Sqh-GFP, (H) UAS-dEGFRλ, UAS-dp110C [file pbio.3000545.s001.tif]

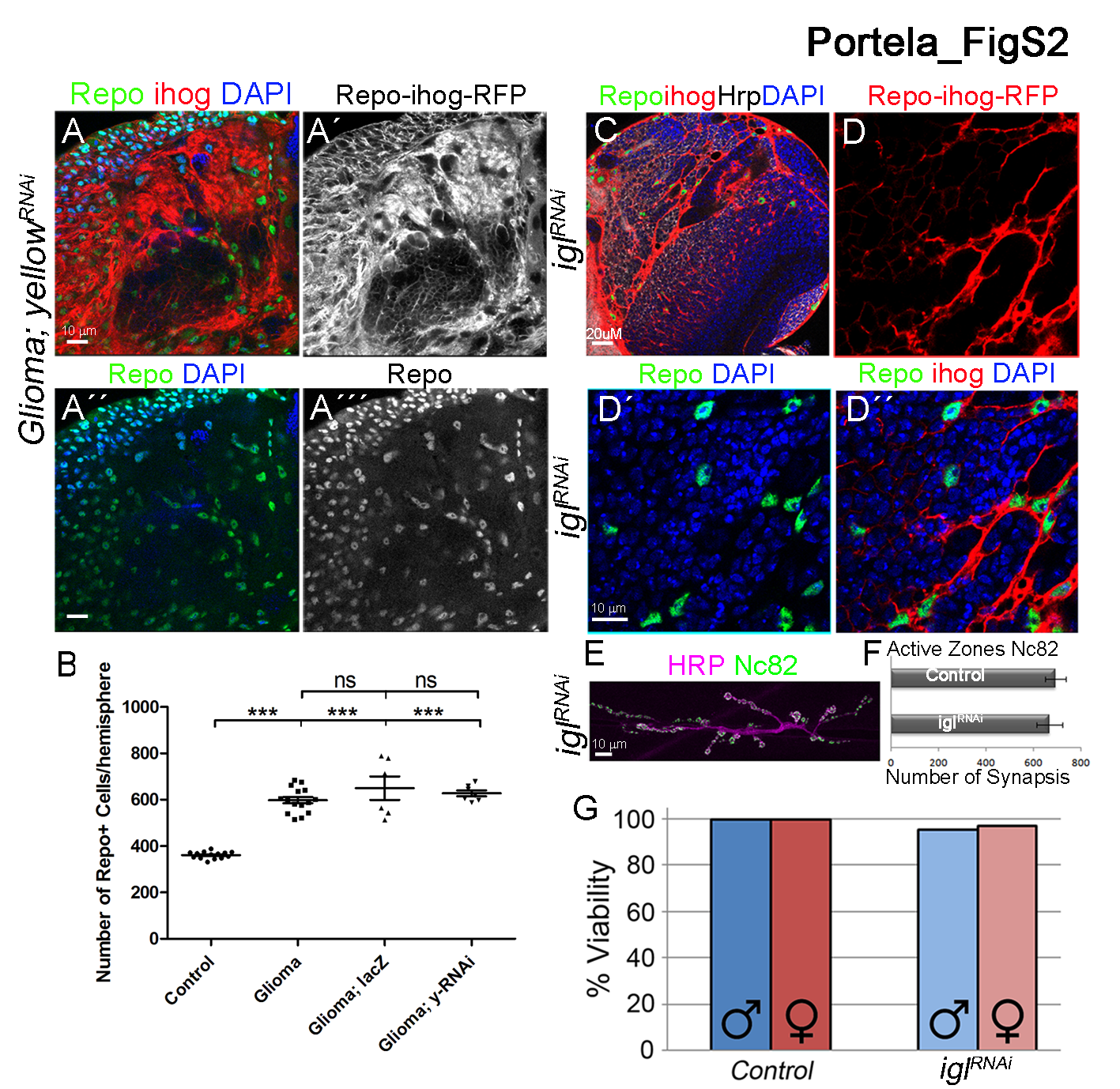

Supplement: S2 Fig — (A–B) Glial network is marked with ihog-RFP (gray or red in the merge). Glial cells are stained with Repo (gray or green in merge), and the number of glial cells are quantified in the following genotypes: Control, Glioma showing an increase in Repo+ cells, Glioma;lacZ, and Glioma;yellow-RNAi showing a similar number of Repo+ cells to Glioma alone. (C–D) Upon igl knockdown by RNAi in normal brains, the glial network (red) is similar to the control. Glial cells are marked by Repo in green. Nuclei are marked by DAPI. (E–F) Neurons (Hrp, magenta) from the larval neuromuscular junction are stained with Nc82 showing the synaptic active sites (green). Upon knockdown of igl, the number of synapses marked by Nc82 (green) is similar to the control. (F) Graph showing the quantification of the synapse number. (G) A viability assay shows that the knockdown of igl does not alter the percent of viability of male and female flies. Error bars show SD; ***P < 0.0001 or ns for nonsignificant. The data underlying this figure can be found in S1 Data. Genotypes: (A) UAS-dEGFRλ, UAS-dp110CAAX; UAS-yellow-RNAi; repo-Gal4, UAS-ihog-RFP, (B) 1. w; repo-Gal4, ihog-RFP/UAS-lacZ 2. UAS-dEGFRλ, UAS-dp110CAAX; repo-Gal4, UAS-ihog-RFP 3. UAS-dEGFRλ, UAS-dp110CAAX; UAS-lacZ; repo-Gal4, UAS-ihog-RFP 4. UAS-dEGFRλ, UAS-dp110CAAX; UAS-yellow-RNAi; repo-Gal4, UAS-ihog-RFP, (C-D) w; repo-Gal4, UAS-ihog-RFP/UAS-igl-RNAi, (E-F) 1. w; UAS-CD8-GFP; D42-Gal4/UAS-igl-RNAi 2. w; UAS-CD8-GFP; D42-Gal4/UAS-lacZ, (G) 1. w; repo-Gal4, UAS-ihog-RFP/UAS-lacZ 2. w; repo-Gal4, UAS-ihog-RFP/UAS-igl-RNAi. Hrp, horseradish peroxidase; igl, igloo; ihog, interference hedgehog; NMJ, neuromuscular junction; RFP, red fluorescent protein. (TIF) [file pbio.3000545.s002.tif]

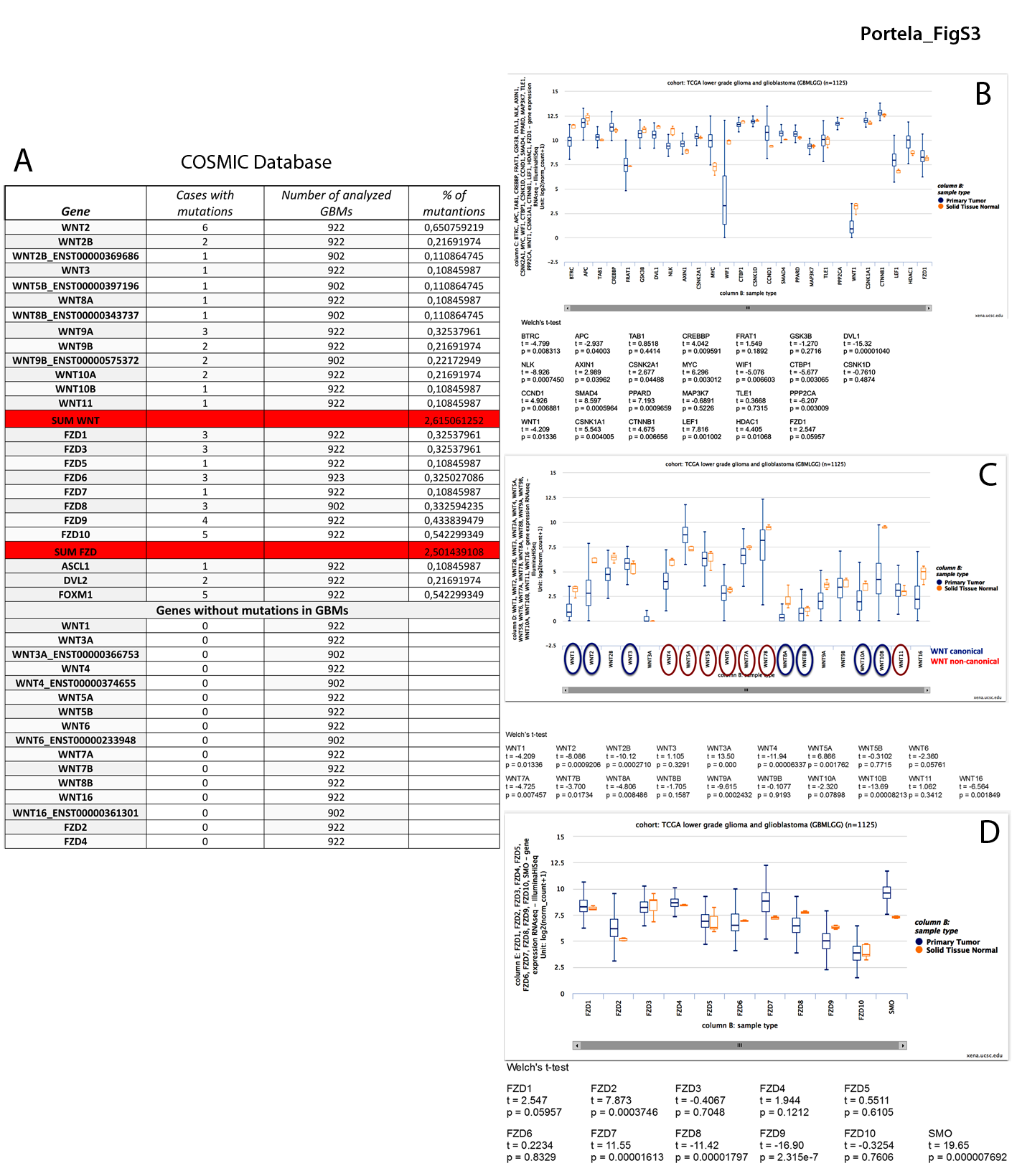

Supplement: S3 Fig — Complete analysis of mutations in human GB samples from COSMIC database http://cancer.sanger.ac.uk/cosmic (A) and TCGA databases through the Xena Functional Genomics Explorer (https://xenabrowser.net/) for transcriptional targets of WNT pathway (B), WNT ligands (C), and FZD receptors (D); data are represented in percentage out of 902 or 922 samples. The total number of cases with mutations in any WNT or FZD gene is shown in red. Genes from WNT and FZD family without mutations in GBs is shown in the bottom. (A) The COSMIC database revealed no alterations in WNT1, WNT3A, WNT6, or WNT5A genes, and 6 cases of human GB cases showed mutations in WNT2 (0.6%). We did not find any case of a GB patient with a gain of expression in FZD2 or FZD4, and 4 cases (0.4%) showed a mutation for FZD9. The total number of mutations related with WNT or FZD genes accounts for 5% of the total GB samples analyzed. (B–D) Analysis of expression levels in primary GB and nontumoral tissues; transcriptional targets for WNT pathway (B) are up-regulated in GB samples. WNT ligands from the canonical WNT pathway are not up-regulated (C) and among FZD receptors, only FZD7 shows significant changes in GB tissue (D). FZD, Frizzled; GB, Glioblastoma; TCGA, The Cancer Genome Atlas; WNT, wingless-related integration site. (TIF) [file pbio.3000545.s003.tif]

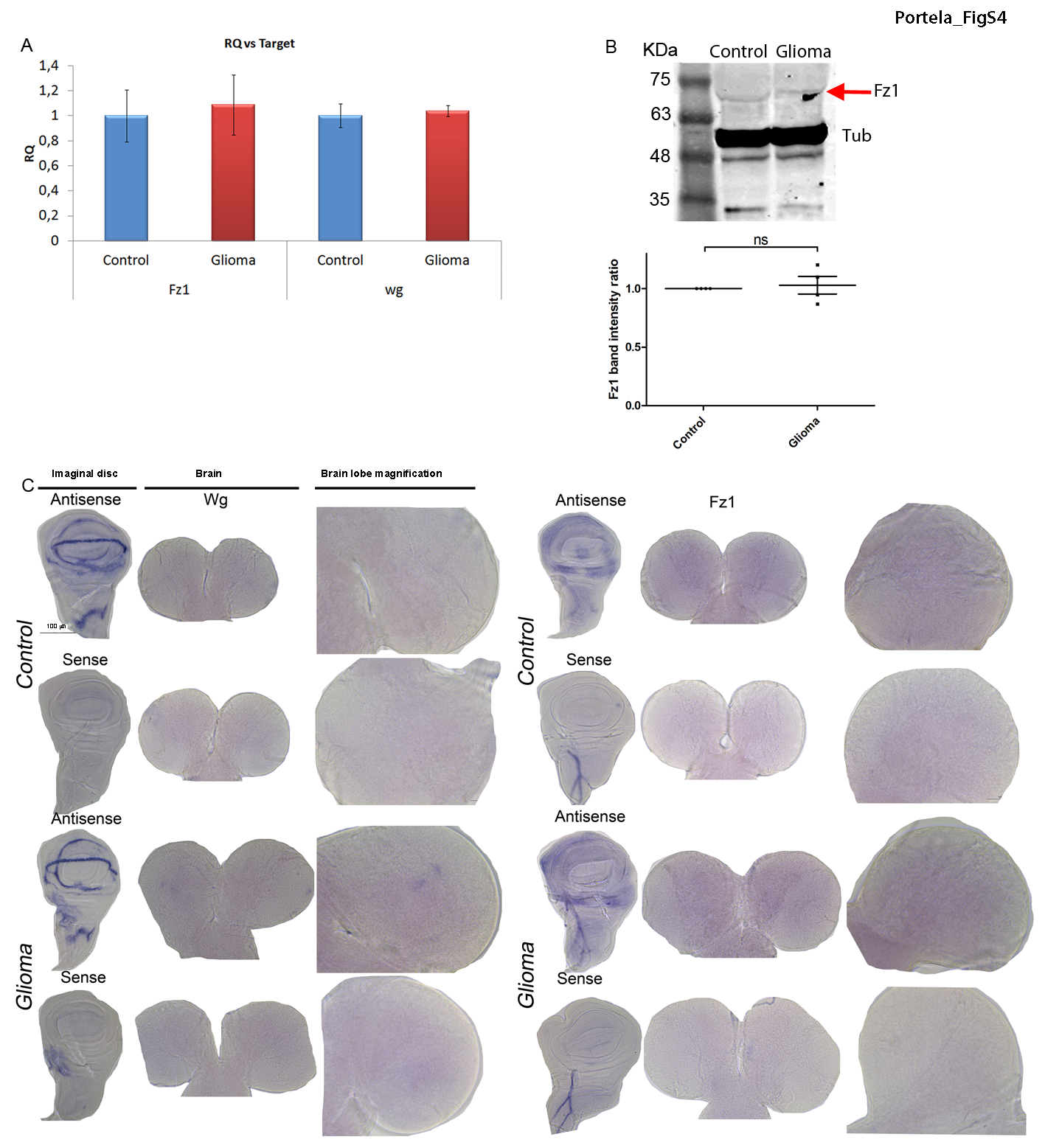

Supplement: S4 Fig — (A) qPCRs with RNA extracted from control and glioma larvae showing no change in the transcription (mRNA levels) of wg or fz1. (B) Western blot of samples extracted from control and glioma larvae showing no change in the amount of Fz1 protein. Error bars show SD, ns for nonsignificant. (C) In situ hybridization experiments for Wg and Fz1 in controls and gliomas showing no change in the transcription (mRNA levels) of wg or fz1. The data underlying this figure can be found in S1 Data. Genotypes: (A-C) 1. w;; repo-Gal4, ihog-RFP/UAS-lacZ 2. UAS-dEGFRλ, UAS-dp110CAAX;; repo-Gal4, UAS-ihog-RFP. Fz1, Frizzled1; qPCR, quantitative Polimerase Chain Reaction; Wg, wingless. (TIF) [file pbio.3000545.s004.tif]

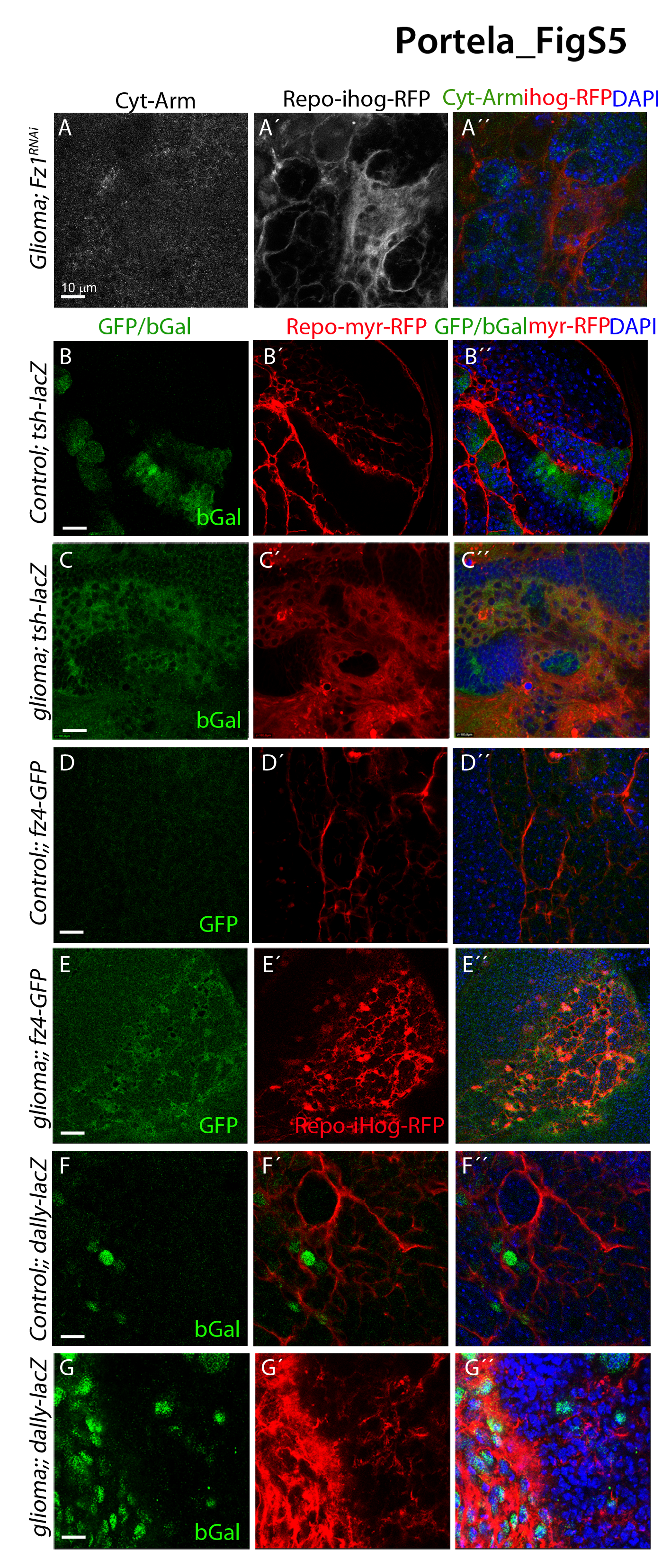

Supplement: S5 Fig — (A) Larval brain sections with glial network labeled in gray (red in the merge) and stained with Cyt-Arm (gray or green in the merge). Knockdown of Fz1 in glioma brains showing a homogeneous Cyt-Arm distribution similar to the control. Quantification of Cyt-Arm staining ratio between Ihog+ and Ihog− domains is shown in principle Fig 5D. (B–G) Glial cell bodies and membranes are labeled with myrRFP or ihog-RFP (red) driven by repo-Gal4. Wg signaling pathway reporters tsh-lacZ stained with anti-bGal (green) (B–C), fz4-GFP in green (D–E), and dally-lacZ stained with anti-bGal (green). (C, E, G) Activation of the Wg pathway reporters in GB cells. Genotypes: (A) UAS-dEGFRλ, UAS-dp110CAAX; UAS-Fz1-RNAi; repo-Gal4, UAS-ihog-RFP, (B) w;; repo-Gal4, UAS-myrRFP/tsh-lacZ, (C) UAS-dEGFRλ, UAS-dp110CAAX;; repo-Gal4, UAS-myrRFP/tsh-lacZ, (D) w;; repo-Gal4, UAS-myrRFP/ fz4-GFP, (E) UAS-dEGFRλ, UAS-dp110CAAX;; repo-Gal4, UAS-myrRFP/ fz4-GFP, (F) w;; repo-Gal4, UAS-myrRFP/ dally-lacZ, (G) UAS-dEGFRλ, UAS-dp110CAAX;; repo-Gal4, UAS-myrRFP/ dally-lacZ. bGal, beta-galactosidase; Cyt-Arm, cytoplasmic-armadillo; Ihog, interference hedgehog; myrRFP, myristoilated red fluorescent protein; Wg, wingless. (TIF) [file pbio.3000545.s005.tif]

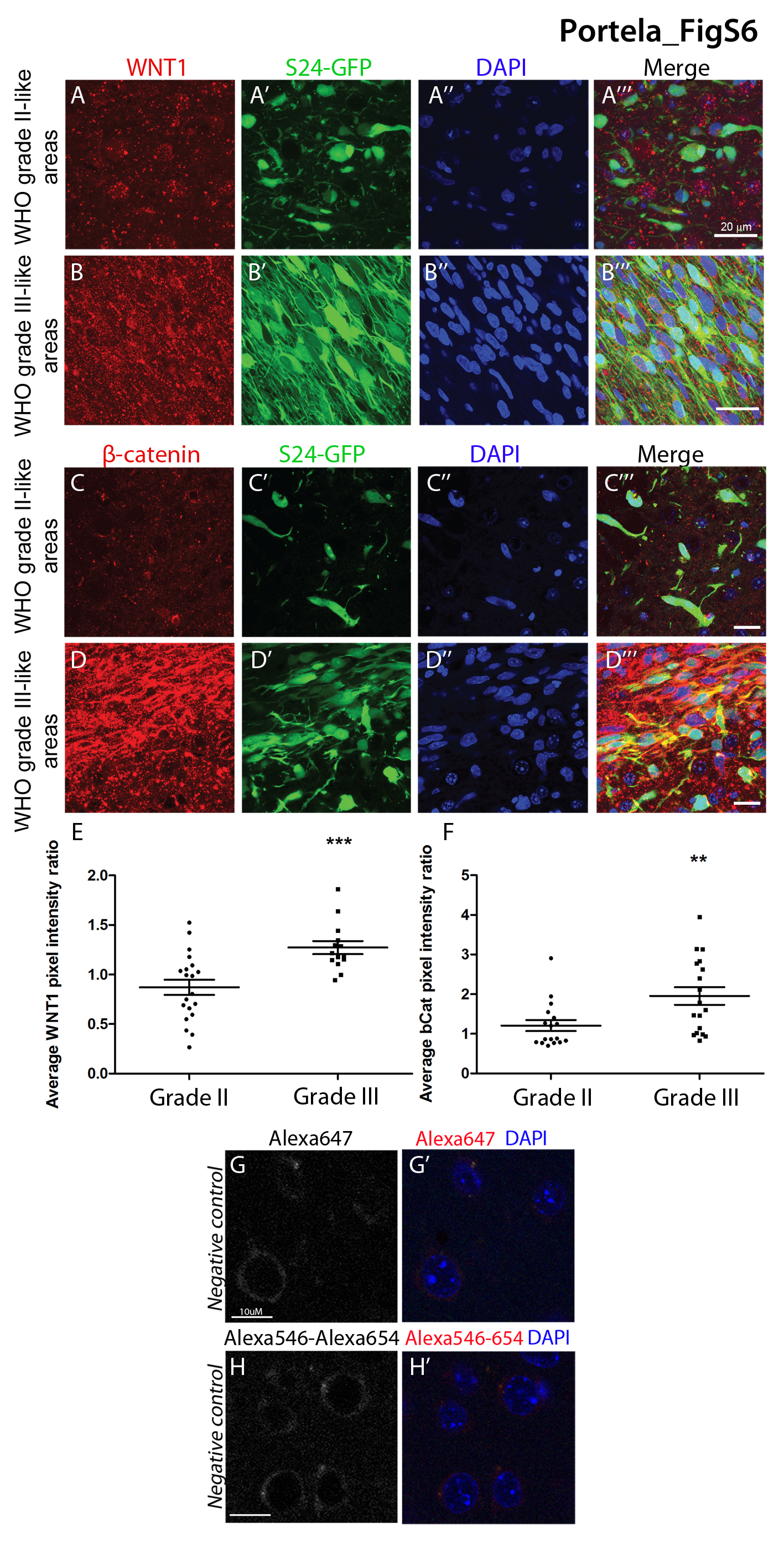

Supplement: S6 Fig — (A–D) A series of grade II and III GB images from S24 xenografts brain sections stained with WNT1 and ß-Catenin (red) show an increase of these signals in grade III when compared with grade II brain sections, indicating that the accumulation of WNT1 and ß-Catenin correlates with the progression of the GB, quantified in panels E–F. (G–H) Technical immunohistofluorescence negative control in NMRI nude control mice brains stained only with the corresponding secondary antibodies showing the background unspecific signal. Nuclei are marked by DAPI (blue). The data underlying this figure can be found in S1 Data. GB, glioblastoma; Wg, wingless; WNT, wingless-related integration site. (TIF) [file pbio.3000545.s006.tif]

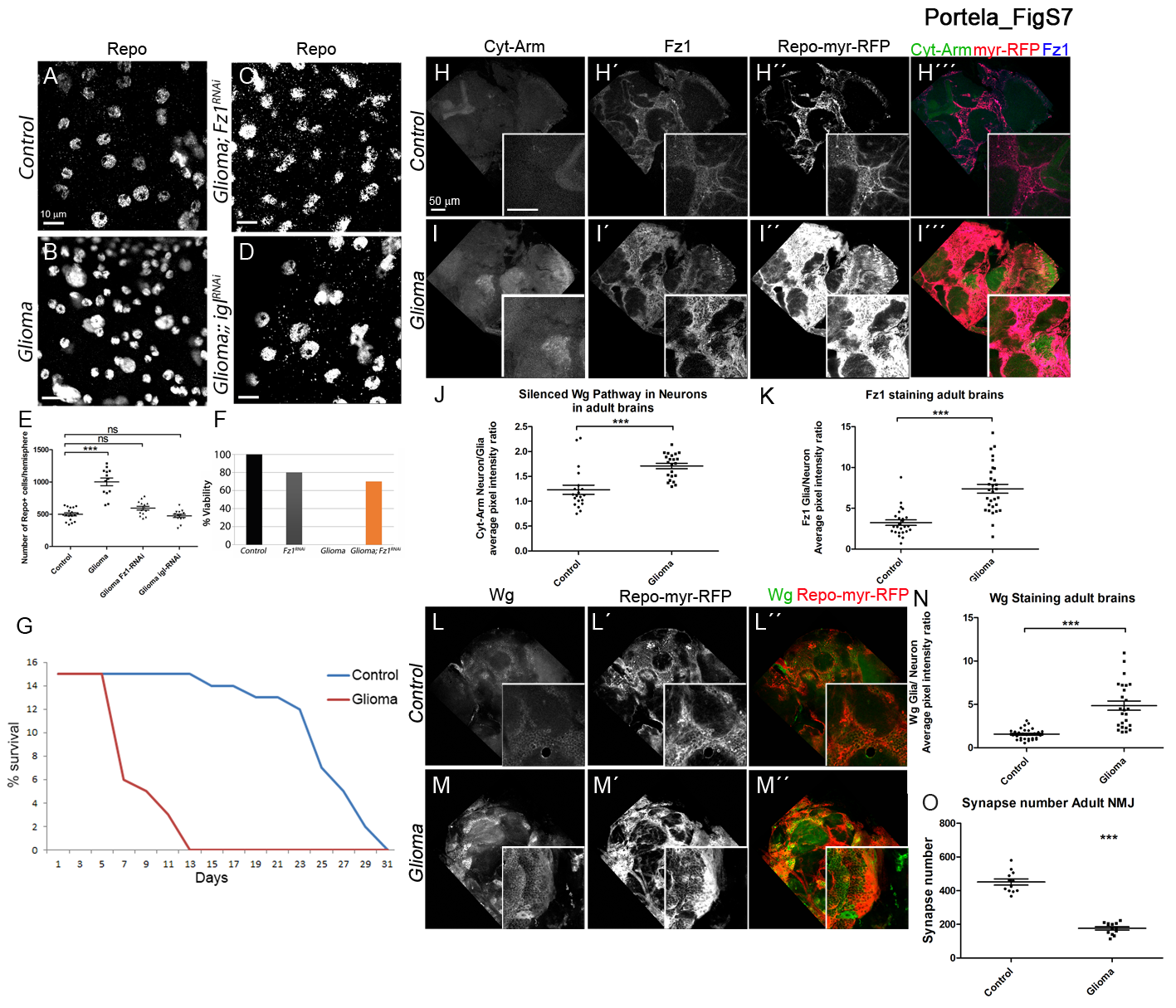

Supplement: S7 Fig — (A–D) Larval brain sections with glial cell nuclei stained with Repo (gray). The number of glial cells is quantified in the following genotypes: (A) Control, (B) Glioma showing an increase in Repo+ cells. (C) Upon knockdown of Fz1 in glioma brains, the number of glial cells is partially restored. (D) Knockdown of igl in glioma cells restores the number of glial cells similar to the control. (E) Quantification of the number of Repo+ cells. (F) Viability assay showing the percental of lethality induced by the glioma that is partially rescued upon knockdown of fz1. (G) Survival curve of adult control or glioma flies after a number of days of glioma induction and progression. (H–N) Adult brain sections 7 days after glioma induction with glial cells are labeled with UAS-myr-RFP (gray or red in the merge) to visualize the glial network and stained with Cyt-Arm (gray or green in the merge), Fz1 (gray or blue in the merge), and Wg (gray or green in the merge) antibodies. (H–J) Cyt-Arm staining specifically marks the mushroom, and it is homogeneously distributed in the rest of the brain tissue in control sections and accumulates in the neurons cytoplasm where it is inactive in glioma brains. Quantification of Neuron/Glia Cyt-Arm staining ratio between RFP+ and RFP− domains (J). (Hʹ–Iʹ, K) Fz1 staining show homogeneous localization in the control brains (Hʹ) in blue. In the glioma brains, Fz1 accumulates in the glial transformed cells (Iʹ), Glia/Neuron Fz1 average pixel intensity ratio quantification is shown in (K). (L–N) Wg is homogeneously distributed in control brains, with a slight accumulation in the RFP+ structures. Wg accumulates in the glioma network similar to the larval brains. Glia/Neuron Wg average pixel intensity ratio quantification is shown in (N). (O) Graph showing synapse number quantification of adult NMJs from control flies and glioma-bearing flies. Error bars show SD; ***P < 0.0001 or ns for nonsignificant. The data underlying this figure can be found in [file pbio.3000545.s007.tif]

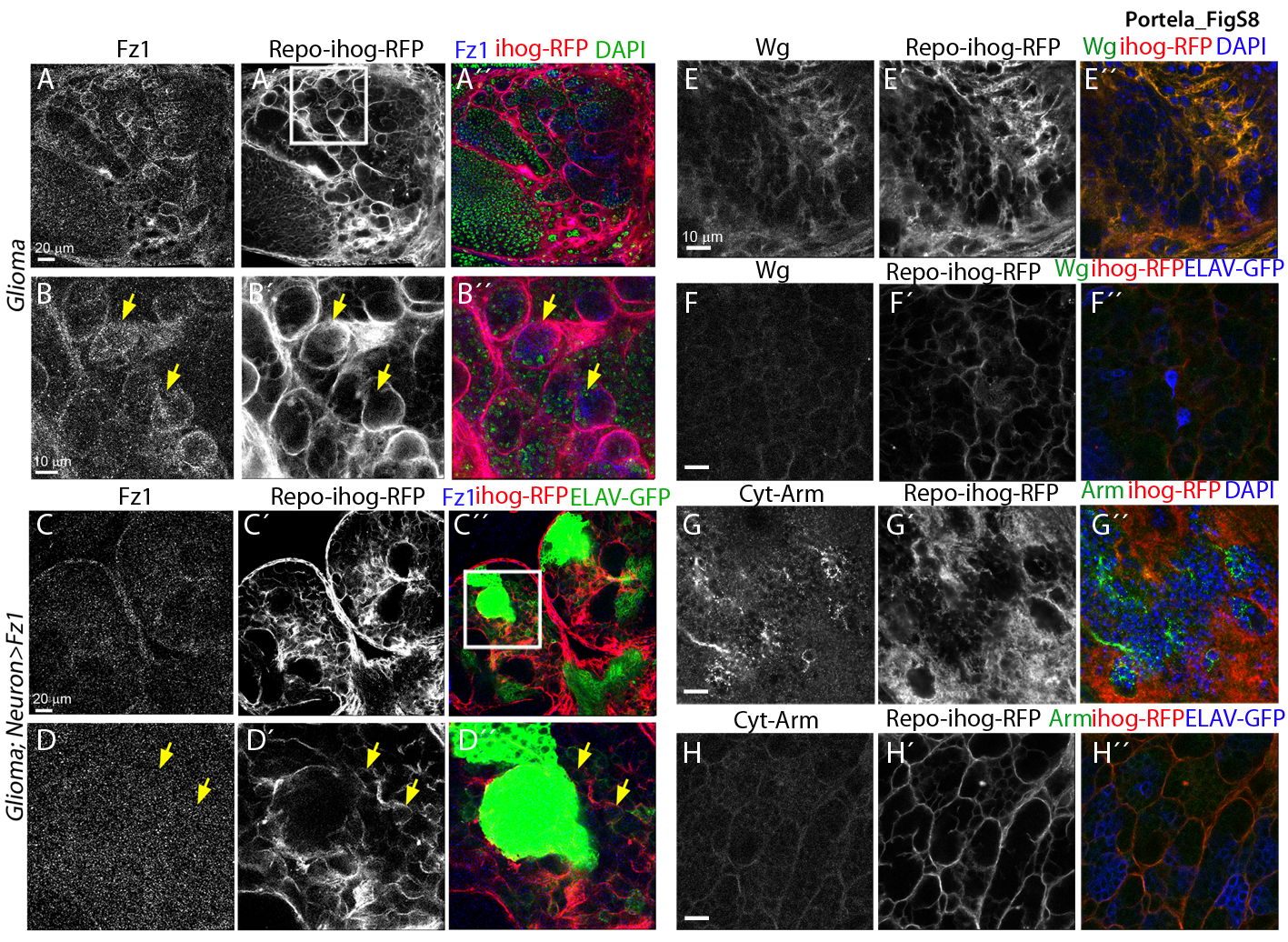

Supplement: S8 Fig — (A–B) Larval brain sections with glial network labeled with UAS-Ihog-RFP (gray or red in the merge) and stained with Fz1 (gray or blue in the merge). Nuclei are marked with DAPI (green). (C–D) Larval brain sections with glial network labeled with UAS-Ihog-RFP (gray or red in the merge). Neurons are labeled with lexAop-CD8-GFP (green) driven by elav-lexA. Fz1 overexpression in neurons restore homogeneous Fz1 protein distribution (gray or blue in the merge) in the brain, rescue brain size, and neuron distribution (panel C and magnification in panel D) compared to panel A and magnification in panel B where the elav-lexA is not present in the glioma brains. Arrows indicate Fz1 staining in the glial membranes at the Glia-neuron interphase of glioma brains and its restored localization in panels C–D. (E–H) Brains from third instar larvae displayed at the same scale. Glia is labeled with UAS-Ihog-RFP (gray or red in the merge) driven by repo-Gal4 to visualize active filopodia in glial cells and stained with Wg or Cyt-Arm (gray or green in the merge). Neurons are labeled with lexAop-CD8-GFP driven by elav-lexA (blue). Fz1 overexpression in neurons restore homogeneous Wg (gray or green in the merge) (F) and Cyt-Arm (H) protein distribution (gray or green in the merge) in the brain, compared with panels E and G where the elav-lexA is not present in the glioma brains. Nuclei are marked by DAPI (blue) in panels E and G. Genotypes: (A, C, E, G) UAS-dEGFRλ, UAS-dp110CAAX; lexAop-Fz1; repo-Gal4, UAS-ihog-RFP, (B, D, F, H) UAS-dEGFRλ, UAS-dp110CAAX; lexAop-Fz1/ elav-lexA, lexAop-CD8-GFP; repo-Gal4, UAS-ihog-RFP. Cyt-Arm, cytoplasmic-armadillo; Fz1, frizzled1; Wg, wingless. (TIF) [file pbio.3000545.s008.tif]

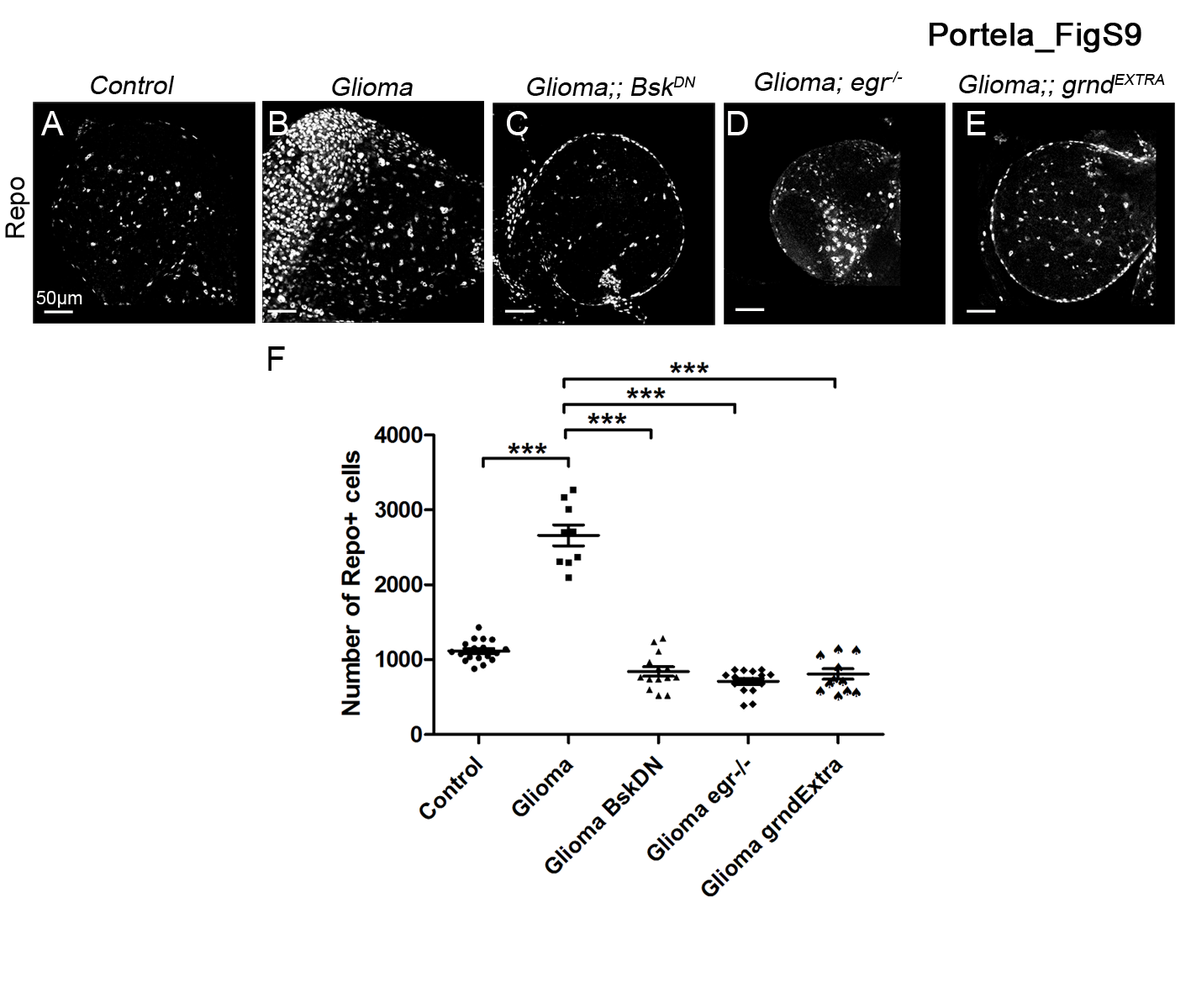

Supplement: S9 Fig — Brains from third instar larvae displayed at the same scale and stained with Repo (gray) in the following genotypes (A–E) control, glioma, glioma bskDN, Glioma egr−/−, and glioma grndEXTRA brain sections. The number of Repo+ cells is quantified in panel F. Error bars show SD; *P < 0.01, **P < 0.001, ***P < 0.0001, or ns for nonsignificant. Scale bar size are indicated in this and all figures. The data underlying this figure can be found in S1 Data. Genotypes: (A) repo-Gal4, ihog-RFP/UAS-lacZ, (B) UAS-dEGFRλ, UAS-dp110CAAX;; repo-Gal4, UAS-ihog-RFP, (C) UAS-dEGFRλ, UAS-dp110CAAX;; repo-Gal4, UAS-ihog-RFP/ UAS-bskDN, (D) UAS-dEGFRλ, UAS-dp110CAAX; egr-/egr-; repo-Gal4, UAS-ihog-RFP, (E) UAS-dEGFRλ, UAS-dp110CAAX; UAS-grndEXTRA/repo-Gal4, UAS-ihog-RFP. GB, glioblastoma; JNK, cJun N-terminal kinase. (TIF) [file pbio.3000545.s009.tif]

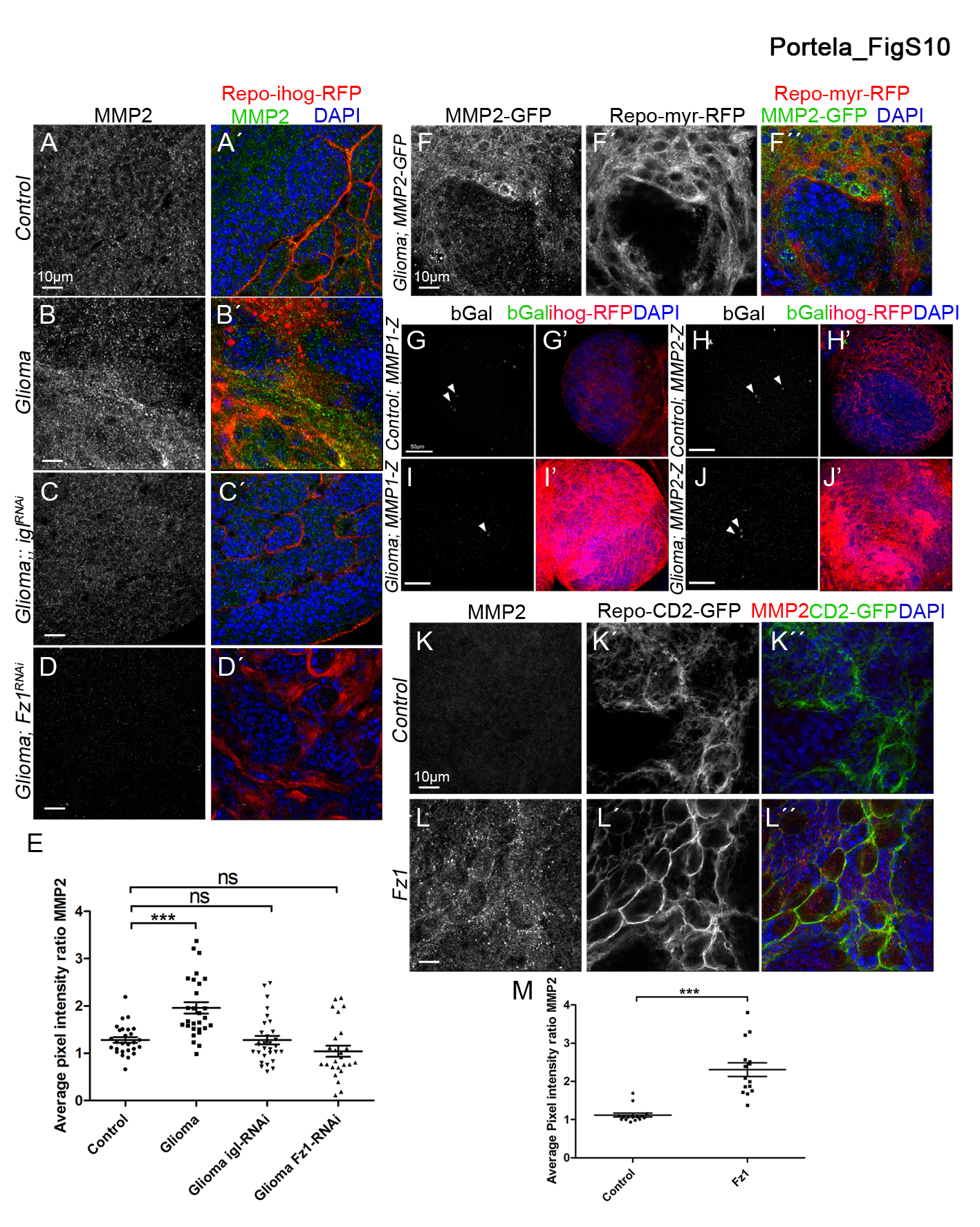

Supplement: S10 Fig — Brains from third instar larvae displayed at the same scale. Glia is labeled with UAS-Ihog-RFP (red) driven by repo-Gal4 to visualize active cytonemes/TM structures in glial cells and stained with MMP2 (gray or green in the merge). (A) MMP2 is homogeneously distributed in control sections, with a slight accumulation in the Ihog+ projections (B) MMP2 accumulates in the TMs and specifically in the projections that are in contact with the neuronal clusters. (C) Inhibition of Gap43 by RNAi in glioma brains restores a normal glial network and MMP2 does not accumulate, showing a homogeneous staining along the brain section. (D) Inhibition of Fz1 by RNAi in glioma brains restores a normal MMP2 distribution. MMP2 does not accumulate showing a homogeneous staining along the brain section. Nuclei are marked with DAPI (blue). (E) Quantification of MMP2 staining ratio between ihog+ and ihog−domains. (F) MMP2-GFP reporter (gray or green in the merge) showing activation in the glioma cell membranes (gray or red in the merge). (G–J) Maximal projections of control and glioma brains with glial cells labeled with UAS-Ihog-RFP (red) to visualize the glial network and stained with anti-bGal (gray or green in the merge) to visualize MMP1-lacZ (G, I) or MMP2-lacZ activity (H–J). (G–H) Control brains show few cells with MMPs transcriptional reporters activated in glial cells (arrowheads). (I–J) In glioma brains, there is a similar activation of MMPs reporters, only few cells in glioma cells (arrowheads). (K–M) Glial cell bodies and membranes labeled by CD2-GFP (gray or green in the merge) driven by repo-Gal4 to the glial cells and stained with MMP2 (gray or red in the merge). (K) MMP2 is homogeneously distributed in control sections. (L) MMP2 accumulates in the glial cells upon Fz1 overexpression. (M) Quantification of MMP2 staining ratio between GFP+ and GFP−domains. Nuclei are marked with DAPI (blue). Error bars show SD; *P < 0.01, **P < 0.001, ***P < 0.0001, or ns for nonsignificant. S [file pbio.3000545.s010.tif]

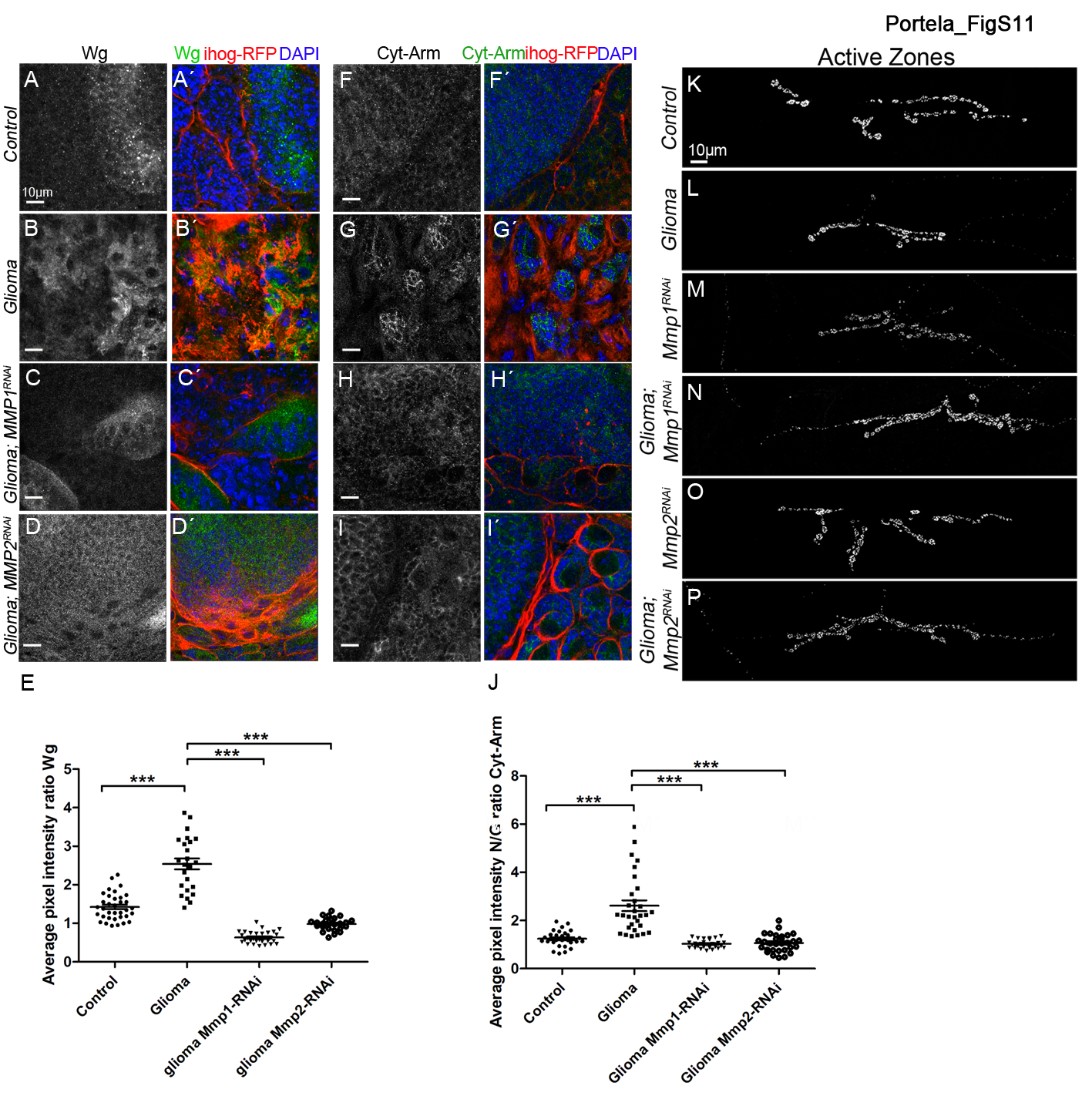

Supplement: S11 Fig — Brains from third instar larvae displayed at the same scale. Glia are labeled with UAS-Ihog-RFP (red) driven by repo-Gal4 to visualize active cytonemes/TM structures in glial cells and stained with Wg (A–D) or Cyt-Arm (F–I) in gray (green in the merge) in the following genotypes control, glioma, glioma MMP1-RNAi, and glioma MMP2-RNAi brain sections. (E) Quantification of Wg average pixel intensity staining ratio between ihog+ and ihog−domains. (J) Quantification of Cyt-Arm average pixel intensity neuron/glia ratio between ihog− and ihog+ domains. Nuclei are marked with DAPI (blue). (K–P) Neurons from the larval neuromuscular junction are stained with Nc82 (brp) showing the synaptic active zones in gray. Upon glioma induction (L), the number of synapses (gray) is reduced when compared with the control (K). The number of synapses is restored upon knockdown of MMP1 or MMP2 (N, P). The quantification of the number of synaptic active zones in all genotypes is shown in Fig 10J. Error bars show SD; *P < 0.01, **P < 0.001, ***P < 0.0001, or ns for nonsignificant. Scale bar size is indicated in this and all figures. The data underlying this figure can be found in S1 Data. Genotypes: (A, F, K) repo-Gal4, ihog-RFP/UAS-lacZ, (B, G, L) UAS-dEGFRλ, UAS-dp110CAAX;; repo-Gal4, UAS-ihog-RFP, (M) UAS-MMP1-RNAi; repo-Gal4, ihog-RFP, (C, H, N) UAS-dEGFRλ, UAS-dp110CAAX; UAS-MMP1-RNAi; repo-Gal4, UAS-ihog-RFP, (O) UAS-MMP2-RNAi; repo-Gal4, ihog-RFP, (D, I, P) UAS-dEGFRλ, UAS-dp110CAAX; UAS-MMP2-RNAi; repo-Gal4, UAS-ihog-RFP. Cyt-Arm, cytoplasmic-armadillo; GB, glioblastoma; ihog, interference hedgehog; MMP, matrix metalloproteinase; TM, tumor microtube; Wg, wingless. (TIF) [file pbio.3000545.s011.tif]
