# Supplementary material for: Glioblastoma cells vampirize WNT from neurons and trigger a JNK/MMP signaling loop that enhances glioblastoma progression and neurodegeneration
Source: PLoS Biol. 2019 Dec 17;17(12):e3000545. doi: 10.1371/journal.pbio.3000545 (PMC6917273; doi:10.1371/journal.pbio.3000545)
Supplement: S1 Raw images — (PDF) [file pbio.3000545.s019.pdf]

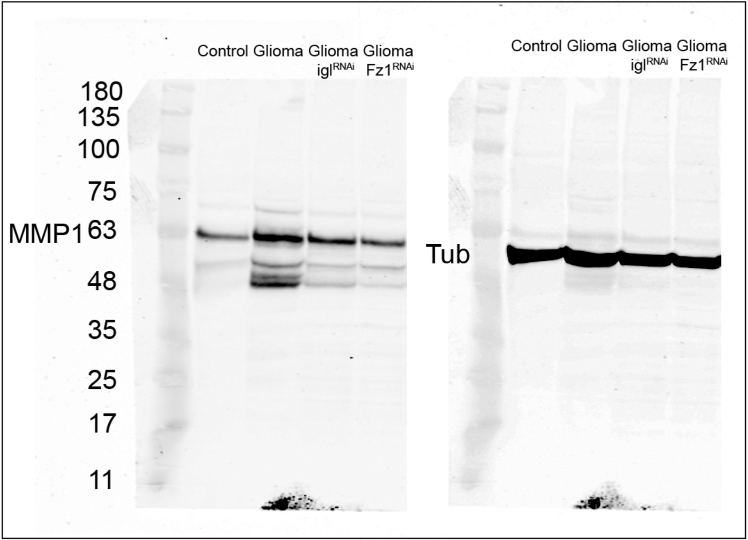

Control
Glioma
Glioma igl RNAi
Glioma Fz1 RNAi
180
135
100
75
MMP1
63
48
35
25
17
11
Control
Glioma
Glioma igl RNAi
Glioma Fz1 RNAi
Tub

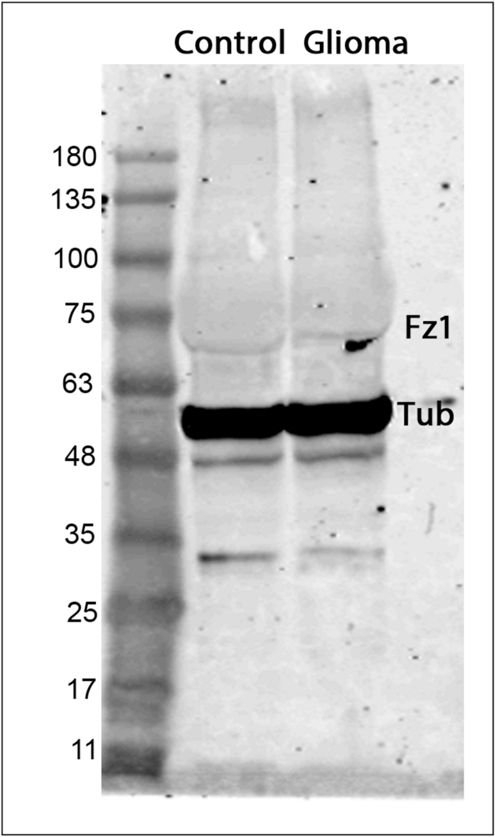
Control
Glioma
180
135
100
75
63
48
35
25
17
11
Fz1
Tub
