# Supplementary material for: Glioblastoma cells vampirize WNT from neurons and trigger a JNK/MMP signaling loop that enhances glioblastoma progression and neurodegeneration
Source: PLoS Biol. 2019 Dec 17;17(12):e3000545. doi: 10.1371/journal.pbio.3000545 (PMC6917273; doi:10.1371/journal.pbio.3000545)
Supplement: Attachment — Submitted filename: Answer to reviewers.docx [file pbio.3000545.s020.docx]

*PBIOLOGY-D-19-00067R1*

*“WNT vampirization by glioblastoma leads to tumor growth and neurodegeneration“*

*Reviewers’ comments*

*Rev. 1:*

*I saw this article as mostly a copy of the article presented by the same first author and last author as PBIOLOGY D19-00117R1. In details looking to these findings it looks like the authors underline here the importance of Fz1 and Wg signalling with modification on assumptions as rapresented mainly here. Why this matter?*

**The importance of Fz1 and Wg signaling matters here because there is little knowledge of the biology underlying GB progression and maintenance. The primary causes of GB are well studied, however, treatments against the founder mutations have failed as described in the introduction.**

**As a consequence, there are no effective therapies against GB. Moreover, the source of WNT/Wingless is relevant as it was not clear in the literature regarding GB how Wg signaling was activated in GB cells. Our data show that Fz1 in GB cells mediates the activation of the pathway in tumoral cells but, the ligand is produced in the neurons which suffer the depletion of Wg by GB cells.**

*This is very puzzling data, in a sense that it should be combined in the other manuscript 00117R1 to make an unique story. Jumping from Flyes to Human is not a simple approach and this make the reviewer upset. As an example , I saw figure Fig.7 (in larval neuromuscular junction stained with Nc82) as similar to data presented in Fig.9 (the active zone) as similar to in the other manuscript 117R1 with additional markers: the authors should have this combined in an unique manuscript to make a more comphrensive manuscript and unified story.*

**We believe that there is a misunderstanding here, Fig 7A-D (67R1) shows neuromuscular junctions with the staining of brp/nc82 (active zones) after wg or Fz1 depletion in neurons. And in Fig 7I-L, it shows the reduction of active zones number caused by GB and the rescue of active zones in GB samples upon Fz1 overexpression in neurons. In Fig 9 (117R1) we show neuromuscular junctions with the staining of brp/nc82 (active zones) but, in this case we evaluate a completely different concept: the rescue of active zones in neurons upon MMPs silencing in GB cells. Both figures include their controls, done in parallel with each experimental set, but we do not find any plagiarism. Thus we believe that the interpretation of the reviewer is not correct. Anyway, we have completely reorganized the manuscript including the order of the Figures.**

*The authors later can rebut to my observations saying that in this two manuscript there are differences, I saw*

*substantially a similar message and I am convinced that can be combined in an unique manuscript. Having saying that reading the two manuscript I feel this format (this version) is much better.*

**67R1 is a study about signals between Glioma cells and neurons. We show that the GB cells expand a network of tumor microtubes (TMs) that enwrap the neurons, accumulate Fz1 and hijack neuronal Wg resulting in tumor progression and loss of synapses in the neighboring neurons.**

**117R1 this second manuscript is about the signaling mechanisms that regulate the expansion of the glioma TM network to infiltrate the brain. And our data show that the expansion of the TMs is regulated by a positive feedback loop including Wg signaling which activates JNK pathway in GB cells, this results in the upregulation of MMPs promoting TM expansion and further activation of the Wg pathway in tumor cells.**

**Anyway we have followed the reviewer´s indications and we have combined both manuscripts**

*In conclusion I am willing to see an unified version of the manuscripts of this 0067R1 manuscript with those presented ni 00117R1 if the authors decide to combine it, otherwise this manuscript is only a partial copy of the other containg less information and containing evidence of plagiarism from the previous paper acted by the same authors at least in the person of the first and last author as compared with 00117R1 manuscript, I feel my action at this time do not deserve my further work.*

**We thank the reviewer for his proposal of combining both manuscripts, this new combined version contains the main discoveries from both manuscripts.**

*Rev. 2:*

*In this MS, Portela and cols study the contribution of different elements in the Wg signaling pathway to the formation of gliomas in Drosophila. Even though the this model has been well established, the conclusions presented by the authors are not well supported by the results. The paper is very difficult to understand. It has many missing parenthesis and contains multiple typos. A deep revision of the writing should be made. Many sentences are not properly separated. Eg, (lines 104-106) “Glioblastoma tumor microtubes (TMs) are described in human GB samples as a cell to cell communication system, we describe here comparable structures in Drosophila glioma brain samples.” This should instead read: “Glioblastoma tumor microtubes (TMs) are described in human GB samples as a cell to cell communication system. We describe here comparable structures in Drosophila glioma brain samples.” This is a recurrent mistake that makes difficult the understanding of the contents presented here.*

**We want to thank the reviewer for the comment; we have reviewed thoroughly the writing, grammar and typos accordingly.**

*The results demonstrating that an increase in Fz1 leads to Wg depletion are very weak. They mostly rely on Arm staining and the authors correlate a decrease of Arm signal with an increase of Arm function in the nucleus. This should be shown in a more convincing manner. Besides, the results showing the Wg targets (lines 321-322) are not convincing. They should show better examples and show controls demonstrating that those target genes are sensitive to changes in wg pathway activity (LOF and GOF) in the brain.*

**We have evaluated Wg signaling activity in brain cells, by analyzing Cyt-Arm staining and with the specific reporters shown in figures 3 and S5 including wild type and comparing to GB brain samples. These reporters were initially described in the following papers:**

**Nkd-lacZ: Yang et al 2013 Development**

**Dally-lacZ: Fujise et al 2003 Development or Han et al 2004 Development**

**Tsh-lacZ: Moran et al 2013 PLoS ONE**

**Arm-GFP: Kiger et al 2007 Dev Biol**

**Importantly, we show several functional experiments that strongly support these conclusions:**

1) **After Fz1 knockdown in GB cells, Cyt-Arm is restored to control levels (Figure S5A compared to Figure 3A).**
2) **We also demonstrate that Wg is dispensable in the GB cells because Wg knockdown in GB cells does not affect the increased number of glial cells or the volume of the GB network (Figure 4C compared to 4B and quantified in 4E-F).**
3) **However, we show that reduction of Wg pathway in neurons (Wg and Fz1 knockdown in motor neurons) is sufficient to cause neurodegeneration (Figure 5G-J).**
4) **Moreover, we demonstrate that this is mediated by Fz1 in GB cells. We did this with the following experiment: we generated GB cells and removed Fz1 (Fz1KD) from them and activate Wg pathway downstream (constitutively active Arm(s10)) in the GB cells. The results show that the number of GB cells and the volume of the TMs network remain similar to normal GB brains (Figure 4D compare to 4B and quantified in 4E-F). However, the analysis of the neighboring neurons shows that under these circumstances (Glioma/armS10; Fz1KD) the number of synapses is rescued (Figure 5E compare to 4B and quantified in 4F). This experiment demonstrated that Fz1 accumulation in the GB cells is necessary for the synapse loss in the neurons and we showed that a reduction of Wg pathway in the neurons is responsible of the synapse loss.**
5) **Finally, we showed that if a GB is genetically induced but the neuronal Wg retrieval by GB cells is prevented (by using a membrane anchored Wg specifically anchored only into the neuronal membranes), the GB does not grow (Figure 4G-J), demonstrating that the GB requires neuronal Wg to progress.**

*The authors state “we describe here comparable structures in Drosophila glioma brain samples.” Are TMs and those structures shown in this MS comparable? TMs connect tumor cells. Are those structures also connecting tumor cells. This is a very important point in the MS that should be addressed carefully.*

**We show that glial protrusions form a network in the normal brain (Figure 1D, H, O) and this network expands in GB samples (Figure 1E-G, K-N, P).**

**In addition, EM images to illustrate in detail the TMs (Figure 1K-N). In figure 1L, the TMs from two different GB cells interconnect both cells; we included a description and arrows to exemplify this in this novel version of the manuscript.**

**Moreover, we include a functional experiment showing that *igl/GAP43* Knockdown in GB cells prevents *Drosophila* TMs expansion and tumor progression (Figure S1 J-M, Video S3), similar to the study of TMs in human GB cells where knockdown of human GAP43 was shown to be important for TMs formation and function, and drove TMs dependent tumour cell invasion, proliferation and interconnection (Osswald et al. 2015).**

*The authors do not show evidence proving that that TMs and the structures shown in fly brain tumors are comparable. The authors also claim these structure are comparable to cytonemes but, again, the evidence is very weak.*

**We have dedicated Figure S1 to show that fly TMs can be defined as TMs and also share features with cytonemes. We show that *Drosophila* TMs are positive for the following Cytoneme markers: LifeActin (S1A-C), GMA (S1D), GPI (S1E), and Ihog (1F-G, O-P, S1A-D, S1H-L and throughout the whole manuscript).**

**Moreover, in Oswald et al. 2015 (Nature), human TMs markers are described which also mark fly TMs. The corresponding orthologues are found in human GB TMs human TMs showing several markers: Nestin, actin, Myosin IIa, PDI, and partly positive for β-catenin, β-parvin and GFAP. We have included data in Drosophila using sqh (MLC/Myo-II), MLC (Myosin IIa) and life Actin (Actin). GFAP is a general astrocyte and other CNS cell types cytoskeletal marker, in our case we use the Pan-glial enhancer Repo to mark glial cells in Drosophila. We were not able to test PDI in our Drosophila model since there are no available tools (antibody or reporters) and we tried to stain Drosophila tissues with β-parvin antibody, but it didn’t work in our hands. And Nestin doesn’t have an ortholog in Drosophila.**

**Moreover, we also included functional evidences that these Drosophila TMs require the expression of nrg and igl (Gap43 associated) (Figure S1 H-M), which validate functionally the similarities among Drosophila TMs, human TMs and Drosophila cytonemes.**

*Other concerns are listed below:*

*Fig legend corresponding to Fig 1A-C is confusing. In some cases the panels do not show what is stated in the legends. Eg, (lines 827-828) “both brain hemispheres are enlarged and elongated…”but the Fig shows a single hemisphere. What corresponds to the white square present in in A’ and B’? I suspect are referred to the panels A and B but those tissues are clearly different. Line 829, “shown in A’ and b’” should read “shown in A’ and B’”.*

**We have revised and re-written these figure legends to clarify.**

*Lines 96-100, the authors claim they visualize the volume of the plasma membrane. I guess they refer instead to the area of the plasma membrane (?). Nevertheless, they can’t show volume of anything in 2d pictures as shown in Fig1E, E’. This part is very confusing.*

**We have measured the volume of the plasma membrane; we visualize the glia/GB plasma membrane with the marker ihog-RFP, acquire full stacks of the whole brain lobe with the confocal microscope, and measure the volume of the glia/GB plasma membrane in 3D reconstructions using Imaris (Bitplane), as described in materials and methods. We also included some examples in the supp videos; however, in the figures we just show 2D images to illustrate the biological concept.**

*Lines 101-102, “In addition, we obtained transmission electron microscopy (TEM) images to visualize glia morphology in control samples (Figure 1F-H).” Fig 1H shows a cartoon and not a TEM image.*

**We have corrected this error**

*Line 315, the authors claim they dismantle the glioma network by expressing igl-RNAi. By expressing igl-RNAi, are the authors dismantling the network or preventing its formation? They also claim the network is dismantled by expressing Fz1-RNAi. This is confusing. From Fig 2 I understood that the network is not affected whenFz1-RNAi was expressed in those cells.*

**We corrected the wording of this affirmation, we meant to say “prevent”**

*Lines 401-404, “Moreover, to stress on the contribution of Fz1 receptor as mediator for Wg depletion, we generated glioma cells and silenced Fz1 expression, in addition we expressed a constitutively active form of armadillo (UAS-armS10) to activate Wg pathway downstream Wg-Fz1 in these gliomas”. How can they activate the Wg pathway downstream of Wg-Fz1, if Fz1 is removed by RNAi expression? The MS contain many inconsistencies like this one that must be carefully addressed and fixed.*

**There is another misunderstanding here, this is not an inconsistency. Frizzled 1 is the receptor and, upon Fz1 knockdown, it is possible to activate the Wg pathway downstream by expressing a constitutively active form of arm (*arms10*) which is independent of the activity of Fz1.**

*Even though the topic addressed in this MS is potentially interested, this MS does not reach the standard for publication in Plos Biol. This is mainly due to the very poor presentation and writing, as well as the fact that the conclusions are not well supported by the data.*

**We hope that the clarifications above contribute to reconsider this decision.**

*Rev. 3:*

*Portela et al. utilizes genetic labeling of tumor microtubes (TM) and neuron network to study Wnt/Fz1 signaling in GB. This work “WNT vampirization by glioblastoma leads to tumor growth and neurodegeneration” reveals the mechanism for Wnt/Fz1 signaling activation in glioma cells, mediated through secretable Wnt that induces glioma expansion and neurodegeneration. The term of vampirization was used to describe this phenomenon. The overall findings are interesting and represent a solid work.*

*Although the essential role of Wnt in neurogenesis and Wnt downregulation in neuron degeneration has been reported previously (PMID:23847469, PMID:20409609, PMID:18652670 ), the interaction of glioma cells with neurons by competing Wnt ligand is novel.*

**We want to thank the reviewer for these positive comments**

*The experiments were well designed. The data are solid to support the conclusion. This manuscript is well written and the concept is clearly presented.*

*There are a few minor questions need to be addressed:*

*1. Is Wnt signaling pathway critical for maintaining tumor microtubes (TMs) in glial cells? Figs 2E,F suggest that Fz1 accumulates in glial cells and is localized in TM structure, but knockdown of Fz1 does not disrupt TM. If Wnt signaling is required for glioma growth and TM protrusion, why blocking Wnt signaling by inhibiting Fz1 does not change TM formation?*

**It is difficult to establish a yes or no relation in this specific question. Our data and previous studies suggest that TMs can be initiated by activity of the founder mutations (EGFR and PI3K), already known to be involved in F-Actin polymerization since the downstream effector of the pathway, Akt, phosphorylates many proteins involved in polymerization and stabilization of the actin cytoskeleton (Kakinuma N, et al. 2008, J Cell Biol, Scita G, et al. 2000, EMBO J, Qian Y, et al. 2004, Am J Physiol Cell Physiol, Enomoto A, et al. 2005 Dev Cell). Then TMs reach a source of WNT, the neurons, which boost the expansion of TMs. As we demonstrate in the co-submitted manuscript (PBIOLOGY-D-19-00117R1, now combined with this) Wg, JNK, MMPs and TMs are regulated in a positive feedback loop. Our experiments show that Fz1 knockdown does not fully prevent TMs formation thus, one possibility is that other receptors or parallel signals contribute to TMs formation, since other molecules related to tumor progression have been shown to signal through cytonemes, such as Notch, Hedgehog or TGF (Kornberg 2013 and 2014, Sherer 2008), all this is now included in the discussion.**

**Moreover, It is also important to note that the efficacy of the Fz1-RNAi tool is not great and could be discussed.**

**(A) UAS-Fz1-RNAi tool was validated in epithelial tissue, wing imaginal discs. Upon UAS-Fz1-RNAi expression in the posterior compartment (marked with GFP), there is a reduction in Fz1 protein signal compared with the anterior compartment from the same tissue.**

**However, we have obtained more spectacular results using the membrane anchored Wg in neurons. We show that if a GB is genetically induced but the neuronal Wg retrieval by GB cells is prevented (by using Wg specifically anchored only into the neuronal membranes), the GB does not grow (Figure 4G-J), demonstrating that the GB requires neuronal Wg to progress and suggesting that Wg pathway is important for TMs expansion.**

*2. Does TM structure exists exclusively in human glioma? TM markers GMA, GPI, MLC, Sqh orthologs were detected in Drosophila glioma cells.*

**The corresponding orthologues are found in human GB TMs. In Osswald et al. 2015, there is a characterization of human TMs showing several markers: Nestin, actin, Myosin IIa, , PDI, and partly positive for β-catenin, β-parvin and GFAP. We have included data in *Drosophila* using sqh (MLC/Myo-II), MLC (Myosin IIa) and life Actin (Actin). GFAP is a general astrocyte and other CNS cell types cytoskeletal marker, in our case we use the Pan-glial enhancer Repo to mark glial cells in *Drosophila*. We were not able to test PDI in our *Drosophila* model since there are no available tools (antibody or reporters), and we tried to stain *Drosophila* tissues with β-parvin antibody, but it didn’t work in our hands. And Nestin doesn’t have an ortholog in *Drosophila*.**

**However, we include a functional experiment showing that *igl/GAP43* Knockdown in GB cells prevents *Drosophila* TMs expansion and tumor progression (Figure S1 J-M, Video S3), similar to the study of TMs in human GB cells where knockdown of human *GAP43* was shown to be important for TMs formation and function, and drove TMs dependent tumour cell invasion, proliferation and interconnection (Osswald et al. 2015).**

*3. Since beta catenin nuclear translocation indicates Wnt activation, the percentage of nuclear beta catenin in mouse Xenograft glioma should be quantified in Fig 4I.*

**The quantifications for nuclear beta catenin average intensity in mouse Xenograft glioma compared to controls are included in Fig 3K. Moreover, we show in figure S6 C-D, a series of grade II and III GB images from S24 xenografts brain sections stained with ß-Catenin show an increase of the nuclear signal in grade III when compared with grade II brain sections, indicating that the accumulation of nuclear ß-Catenin correlates with the progression of the GB, quantified in (S6F).**

*4. In mouse Xenograft model, does TM structure form? Neuron markers should be stained to visualize if they are enwrapped with glioma TM. More importantly, where is beta catenin located in neurons? If knockout Frizzled family in S24 cells, does it change beta catenin activation in neurons in this model as seen in Drosophila?*

**TMs in mouse xenograft model were originally described by Dr. Winkler, author of this manuscript (Osswald 2015, Nature). We have a manuscript under consideration describing the physical interactions between GB cells and neurons.**

**Frizzled family in *Drosophila* is composed by 2 members, however, there are more than 10 FZD members in mammals, we are working on the contribution of each single FZD and WNT molecules to define the contribution of each family members, however, the contribution of FZD receptors to GB progression has been already suggested (see** [**Chui-Mian Zeng**](https://www.ncbi.nlm.nih.gov/pubmed/?term=Zeng%20CM%5BAuthor%5D&cauthor=true&cauthor_uid=29789460) **et al. 2018,** [**Int J Mol Sci,**](https://www.ncbi.nlm.nih.gov/pmc/articles/PMC5983605/) **doi:** [**10.3390/ijms19051543**](https://dx.doi.org/10.3390%2Fijms19051543) **).**

*5. Could Wnt1 induce beta catenin activation (by TOPflash analysis) and TM gene expression in S24 cells in vitro?*

**There is abundant literature stating that Wnt1 induces beta catenin activation (translocation) (Chen et al, J Cell Biol 2001; Song et al, Mol Med Rep 2014, reviewed in Alok et al, J Cell Sci 2017 doi: 10.1242/jcs.198093)**

**We are working on a project in collaboration to determine the contribution of each human WNT ligand and each receptor in gliomagenesis. The results indicate that WNT ligands stimulate the formation of projections, compatible with TMs. However, these cells are mutant for additional oncogenes and/or tumor suppressors.**

**To determine the potential induction of projections by WNT, we have overexpressed Wg in glial cells and we cannot see any clear phenotype. These data suggest that WNT can accelerate the formation of TMs but it is not sufficient to induce them.**

*6. In Fig 4E-H and Fig S7B-G, Wg pathway reporters were only co-stained with glia but not with neuron, how was the conclusion drawn that the reporter activities were down in neurons?*

**In both figures, control samples (without GB) are included and arrows indicate the activity of Wg reporters in neurons (Figure 3-E, G and Figure S5 B, D and F). Please take into consideration that in the *Drosophila* model, all glial cells in control and glioma are marked with myr-RFP/Ihog-RFP, so the unmarked remaining cells are neurons.**

*7. Fz1 knockdown in glia cells or Fz1 overexpression in neurons rescues neuron synapse degeneration. How about the behavior change? Is climbing assay possibly measured in those flies?*

**GB animals are rescued in their survival upon *Fz1-RNAi* expression (Figure S7F) but we have not included specific behavioral data as we are writing a manuscript focused on the behavioral impact of GB progression.**  *__________________________________________________*

*PBIOLOGY-D-19-00117R1*

*Reviewers' comments*

*Reviewer #1:*

*I saw this article as mostly a copy of the article presented by the same first author and last author as PBIOLOGY D19-00067R1. In details looking to these findings it looks like the authors underline here the importance of JNK epistatic function to Frz signalling together with MMP1 and MMP2 studies in the same way and using some additional models as presented D19-00067R1 with modification on assumptions as rapresented mainly here. Why this matter? This is very puzzling data, in a sense that it should be combined in the other manuscript 000-67R1 to make an unique story. Jumping from Flyes to Human is not a simple approach and this make the reviewer upset by reading the two manuscripts and seeing duplications in sentences in introduction and conclusions. Then in results, as an example , I saw figure 9 data (the active zone here) as similar to Fig.7 (in larval neuromuscular junction stained with Nc82) in the other manuscript 67R1 with additional markers: the authors should*

*have this combined in an unique manuscript to make a more comphrensive manuscript and unified story. The authors later can rebut to my observations saying that in this two manuscript there are differences, I saw substantially a similar message and I am convinced that can be combined in an unique manuscript.*

*In conclusion I am willing to see an unified version of the manuscripts 00111R1 and 0067R1 if the authors decide to combine it, otherwise this manuscript is only a partial copy of the other containg less information and containing evidence of plagiarism from the previous paper acted by the same authors at least in the person of the first and last author as compared with 0067R1 manuscript, I feel my action at this time do not deserve my further work.*

**This text is identical to the other answer from reviewer 1. Please see previous answer to Rev 1.**

*Rev. 2:*

*Casas lab identify a molecular loop formed by Wg, JNK and MMP1 that is involved in glioma formation if Drosophila melanogaster. The manuscript shows that JNK , Wg, and MMPs are expressed in fly glioblastoma. The expression and relationships of these elements in different fly tumors have been well documented before in numerous papers. In my opinion, these observation lack novelty. It is not surprising that those factors are also present in fly gliomas.*

*In the last part of the manuscript the authors show that MMPs are involved in glioma expansion. This has also previously described in tumors of epithelial origin.*

*In summary, the manuscript provides a new example of a fly tumor where JNK, Mmp1 and Wg are present, but, in my view, lack novelty to reach the threshold required for publication on P Biology.*

**We understand that these relations have been already described in epithelial tumors, but they have not been described in glial cells yet. The mechanisms that facilitate the depletion of factors such as Wingless through the formation and expansion of TMs should be of relevance to understand how gliomas progress and the mechanisms that regulate their infiltration into the brain. Current strategies to prevent GB advance are not efficient, therefore, the novel information that we present here regarding the cellular requirements to promote GB expansion are of great relevance. The existence of a positive feedback loop is a matter of discussion regarding the perpetuation of GB progression, independent of founder mutations (PI3K/EGFR). This aspect is a key aspect in the failure of specific treatments directed against these founder mutations and brings the possibility of other relevant hits as potential targets for GB, this is included in the discussion. However, we have reduced the details regarding this information in the combined manuscript to only 2 figures (Figure 6 and 7).**

*Rev. 3:*

*Portela et al. took advantage of Drosophila model to study the network of Wingless/JNK/MMPs in glioblastoma (GB) with conventional developmental biology methods. GB was induced by constitutive activation of EGFR and PI3K pathways. Their work reveals that Wg signaling activates JNK and induces MMPs, which is determinant in GB progression by facilitating tumor microtubes (TM) infiltration, expansion and neurodegeneration and consequently Wg activation in GB cells as a positive feedback loop. The mechanism for TM expansion regulated by Wg in GB is novel. However, the investigation on Wg, JNK, MMP and TM network identifies each inter-regulation between the genes including Wg, Fz1, Cyt-Arm, MMP1/2, grnd and JNK, leading to confusing signaling crosstalks. The data presented don’t fully support the proposed network in Figure 9H. It would be better to move some figures into the supplementary data, and also to reform the data to more clearly support the model.*

**We have now combined these 2 manuscripts and moved some figures to the supplementary data as suggested by the reviewer, although we have also included some novel data regarding the transcriptional regulation of MMPs in GB (Figure 8E and S10G-J).**

*There are some major concerns as below:*

*1. Glia expansion is induced by EGFR/ PI3K activation in Drosophila GB model. Were all glia cells transformed into tumor cells?*

**Yes, this *Drosophila* model is based on the enhancer “repo” which activate the expression of EGFR and PI3K active forms in all glial cells.**

*2. Wg was upregulated in GB model, suggesting that glia cells acquire stemness. Is Wg expressed in a part or all of the glia cells? Are Wg+ cells stem cells? Are Oct4 and Sox2 expressed in glioma cells?*

**We believe that there is a misunderstanding here. We show that Wg is not upregulated, but re-localized (see figure S6 from manuscript** *PBIOLOGY-D-19-****00067R1,* now S4 in the combined manuscript). The neurons are the source of Wg, and GB cells steal Wg through the Fz1 accumulated in the TMs.**

*3. Grnd is required for Fz1 distribution. Is it dependent on JNK activation? Does JNK overexpression rescue Fz1 distribution in Grnd-/- GB?*

**Grnd is required to activate JNK pathway and MMPs which result in the expansion of the TMs which localize Fz1 to retrieve Wg from the neurons.**

**JNK activity is tightly regulated as it can trigger apoptosis. We have attempted to carry out the suggested experiment, and we first activated JNK (*UAS-Hep^Act^*) in GB and wild type glial cells, but this is lethal in all cases.**

*4. Figure S3 showed expression of Egr ligand and Grnd receptor was increased in GB compared to control brain. Although glia TM structure was highlighted by UAS-Ihog-RFP labeling, it was still hard to see the localization of Egr/ Grnd on neurons or glia. Flow cytometry analysis will be helpful to distinguish Egr/ Grnd expression in each cell types.*

**These experiments were confusing and did not contribute to the line of the manuscript, we have not included these data in the novel combined version.**

*5. Wg activation is accompanied with beta catenin nuclear translocation. Cyt-Arm seems to localize mostly on the membrane in glioma (Figure 6, Figure S7). Does downstream gene expression of Arm change, such as the orthologs of Axin2, Myc or Ccnd1/2?*

**Please find in Figure 3G-H (*nkd-lacZ*) and in FigureS5B-G (*tsh-LacZ, fz4-GFP* and *dally-lacZ*) additional reporters that support the activation of Wg pathway in glioma cells and the repression in neurons.**

**Besides, we have reduced Myc expression in glioma cells (Myc-RNAi) and rescued GB progression and animal viability. Therefore, we conclude that Myc is necessary for glioma progression, these results go in line with Read et al. 2009, PLoS Gen, that already described the activation of dMyc and dCycD, among others, in this *Drosophila* glioma model.**

*6. Fz1 is essential and sufficient to induce MMP1 expression as demonstrated in Figure 1 & 2. Vice versa, MMP1 is required for Fz1 shown in Figure 8. It is not clear if the regulation is on a transcriptional or post-translational level. Is MMP1 mRNA changed or secretion increased? Is Fz1 protein stabilized by MMP1 directly? In situ hybridization may be considered for mRNA detection. RFP+ glia cells could be isolated by flow sorting for in vitro treatment.*

**We have analyzed MMP1 and MMP2 mRNA expression in with transcriptional reporters (*MMP1-LacZ* and *MMP2-LacZ*) and we have not observed any difference in GB samples compared to controls (Figure S10G-J). Moreover, qRT-PCR experiments do not show either a transcriptional upregulation of *MMP1/MMP2* in glioma samples (Figure 8E).**

**Moreover, the results in Figure S4 indicate that Fz1 is not regulated transcriptionally in glioma and we detect comparable protein amount in control and glioma brains, therefore we discuss that Fz1 is re-localized into the TMs surrounding the neurons.**

*7. Since Wg regulates MMP expression, is Wg inhibitor better than MMP knockdown to extend fly survival?*

**We cannot knockdown/knockout or inhibit Wg from neurons as this combination is lethal “per se”, however, we show that *Fz1-RNAi* (Figure S7F) and *MMP1/MMP2 RNAi* (Figure 10I) rescues GB-induced lethality.**

*8. The synapse of neuromuscular junction was detected in glioma. Was the synapse decrease due to neuron apoptosis or plasticity? It needs to be clarified.*

**We analyzed apoptosis in neurons exposed to GB brain and its functional effect.**

**We have done TUNEL experiments and detected an increase of apoptosis in neurons exposed to GB (see Figure for Reviewer below), however, neurons detach from their targets before getting into the apoptotic process which is the last step in a neurodegenerative process. Synapse decrease is an initial step in the neurodegenerative process that precedes the apoptotic process which it is shown in Figure 5A-B.**

*Minor: In Figure2, CD8-GFP was stated in the legend, while CD2-GFP was labeled on top of the images.*

**We have corrected this.**
