# Supplementary material for: Glioblastoma cells vampirize WNT from neurons and trigger a JNK/MMP signaling loop that enhances glioblastoma progression and neurodegeneration
Source: PLoS Biol. 2019 Dec 17;17(12):e3000545. doi: 10.1371/journal.pbio.3000545 (PMC6917273; doi:10.1371/journal.pbio.3000545)
Supplement: Attachment — Submitted filename: Point by point response to reviewers 030919.docx [file pbio.3000545.s021.docx]

Dear Editor,

We are submitting the revised version of the manuscript *PBIOLOGY-D-19-01582R1* ***“*Glioblastoma cells vampirize WNT from neurons and trigger a JNK/MMP signaling loop that enhances glioblastoma progression and neurodegeneration”** The manuscript has been re-written according to the reviewer´s comments, and we have addressed all the concerns raised by the reviewers. Below, you can find a point-by-point response to the points raised by the reviewers, explaining all the changes in the text.

We would like first to thank the reviewers for their positive comments: *“The unified version is much improved. The authors combined two previous manuscripts and re-organized the story, making it more comprehensive. The paper has been improved. Most of the concerns from the last review have been addressed with proper experiments completed. Overall, the quality of the revised manuscript is improved, and a further revised version is in favor of publication”.*

Rev. 1:
The authors has follow reviewer indications. As such the merge the manuscripts informations enhance the manuscript focus and the final information to the readers. Still there are sentencees need explanations. My view is that the manuscript need some additional cleaning avoiding sentences with over hestimation of the model. Jumping to the human Glioma is not an easy task.

We have polished the text to avoid over interpretations and discussed the limits or potential of these results.
There are some sentences thougth I found difficult to judge, based on their observations which are not fully addresses, as such, I suggest the authors to deplete or need data to further confirm in this paper . An alternative is to bounce to human, there are now several geneexpression data signature in glioma that can be used to prove in silico this model.

We have reviewed human databases and included the data in the manuscript, however, we have specified in the text that these results are mostly obtained in *Drosophila* and thus, further experiments in human samples will be required to validate our findings. We propose a mechanism of Wg depletion and the key role of TMs in GB progression that could be relevant for human GB. Up to date the experiments to study glioma and neuron interactions cannot be done in cell culture and *Drosophila* is a suitable animal model to study these relations in depth.

I am not able to judge the fly experiments and the use of all these mutants as presented, I assume they all work perfectly but I am sure some are " leaky mutants "and as such a description of the leakiness should be add in the text in the methology section. Overall there is still some work to be done but the unified version is much improved.

All the tools used in this manuscript have been validated previously by other authors and/or repositories. UAS-RNAi lines from VDRC (https://stockcenter.vdrc.at/) do not have off-targets according to the description in the repositorie. Other UAS lines are referred accordingly to the original publications. In most cases, we have addressed the same biological question using several tools and strategies to reach similar results and therefore, reinforce the conclusions, i.e. JNK signaling is required for GB progression, we tested 5 tools to block the JNK pathway: eiger mutant, Bsk^DN^, grndRNAi, grnd^extra^ and grnd-/- and obtained comparable results.

I found some problems on some sentences in the results section then repeated and followed in discussion that need a peculiar attention.
1) "These results suggest that wg expression in glioma cells is not relevant for glioma progression". Althought the experiments are of interest I don't think this in human is the case, persistent WNT signalling is observed in those uncurable tumours, so this cannot be extrapolated from fly to human and as such the mechainism need to be reevaluated.
We want to thank the reviewer for this comment, as this is one important section of this manuscript and a hot topic of discussion in the field. We have modified this affirmation to leave space for discussion and other interpretations of the results.

As shown in Figure S3, data from two different databases (COSMIC and TCGA) show that:

1- The number of cases with mutations in WNT or FZD genes among 922 human samples is less than 5% in total
2- Expression data (RNA seq) from the TCGA database that among 19 WNT genes analysed, none of the WNT canonical ligands is upregulated in GB and only WNT 5A (non-canonical) shows an upregulation as compared to normal tissue (p=0.0017 Welch´s test, please see Figure S3C).
3- We have now included a publication by Zhang et al. [Oncol Lett](https://www.ncbi.nlm.nih.gov/pmc/articles/PMC5768065/). 2018 Jan; 15(1): 610–617 “Expression profile and clinical significance of Wnt signaling in human gliomas”. This manuscript indicates that: *“...the expression of Wnt2b and 5a, and frizzled 2, 6 and 7 were highly associated with the prognosis of patients with glioma”*, it is important to note that these proteins are not related to the canonical Wnt pathway.
4- In addition, we have analysed a third different database (GEPIA) and we have obtained similar results. According to this database there are no WNT genes upregulated or whose upregulation correlates with GB patient´s survival reduction:

Gene expression:

<http://gepia.cancer-pku.cn/detail.php?clicktag=degenes>

Genes associated with survival in GB:

<http://gepia.cancer-pku.cn/detail.php?clicktag=survival2>

The results obtained from this database go in line with the other two databases already included in the manuscript, therefore we have not included these results as a figure but it could be done if required.

We agree that the results in this manuscript are based on the fly model, therefore these proposed mechanisms to activate WNT signalling in GB cells by depleting WNT from the neurons, will require further confirmation in human samples. We have emphasised the difference between Wg expression and Wg signalling activation for better understanding.

The results suggest that *Drosophila* Wg expression is not relevant in glioma cells but signalling is very relevant (in flies and in humans).

Our aim is not to extrapolate this particular experiment to humans, but to bring new avenues for discussion, thus we have specified in the text that this conclusion refers to the fly model (page 13). Wnt canonical pathway is of great relevance for GB patient´s, therefore, the source of activating ligands of this pathway should be studied in detail.

2)"Altogether, these results reveal a novel positive feedback loop involving Wg, JNK and MMPs that contribute to GB development and progression. These discoveries reveal potential pharmacological targets and novel avenues for the treatment of GB".
Have you consider the crosstalk effects? JNK and MMPs are know to be activated by several other pathways and a crosstalk action of these pathways in human are known to underway to go to a precise dissection.
We understand that the results are obtained mostly in flies and we have indicated it throughout the text. We have included in the discussion the potential relevance for human GB.
TGF.beta/BMP/MAPK as well as PI3K has role on activating JNK and MMP mostly via PTEN inhibition, please follow this and comment it into the manuscript.

# We fully agree with this possibility and we have included this concept in the discussion. BMPs (dpp in flies) signal in neighboring cells via cytonemes and play a role in BMP signaling in epithelial cancer cells (Cytoneme-mediated signaling essential for tumorigenesis. Sol Fereres… Thomas B. Kornberg. <https://doi.org/10.1101/446542>). These results support our hypothesis of the importance of TMs in cancer signaling as a convergence point for various oncogenic signals.

# Regarding PI3K, we cannot exclude that PI3K activates JNK in glial cells but, we have performed experiments to upregulate only PI3K signaling in glial cells and this is not sufficient to form TMs, to upregulate wg signaling, to induce MMP1 accumulation or to stimulate GB progression * (this was previously reported by (Read et al PLoS Gen 2009)). Therefore, we have shown that JNK pathway activation, and MMPs accumulation in consequence, are required for TMs expansion, and viceversa. The mechanisms that lead to the establishment of this loop could be various, including the combined activation of EGFR and PI3K pathways (PTEN inhibition).

# *Please see Figure MMP1 staining in myc Pi3K Egfr

3) Neurodegeneration because of Wg depletion, ....well this is very strong hypothesis. Neurodegeneration it is supposed to start if there is oxidative stress and mithocondrial dysfunction on the other hand WNT signalling loss is causing synapses neurodegeneration in AD (Alzheimer disease), this os only one example --- so this sentence above need a better rephrasing. Maybe you want to investigate oxidative stress in your model and link to neurodegereration as well as to those initiation events of neurodegeneration such as impairing of the autophagy, but at this time the message goes cunfused becouse too many aspects to take in consideration at this time. My suggestion is cut the story to the major findings.
We have evidences of mitochondrial dysfunction in neighbouring neurons which will be included in a future manuscript about the physiological effect of GB in neurons. We aim to focus this current manuscript in the mechanisms that participate in the communication GB-neuron via TMs. So, even though this proposal is very interesting we believe that including mitochondrial dysfunction in the current manuscript would not help to clarify the message of the paper.

To strength our hypothesis we have now included further supporting evidences of Wg signalling role in synapse formation.

For example:

Packard et al 2002 Cell <https://www.ncbi.nlm.nih.gov/pmc/articles/PMC3499980/>

“the Drosophila Wnt, Wingless (Wg), is essential for synapse development. We show that Wg and its receptor are expressed at glutamatergic NMJs, and that Wg is secreted by synaptic boutons. Loss of Wg leads to dramatic reductions in target-dependent synapse formation, and new boutons either fail to develop active zones and postsynaptic specializations or these are strikingly aberrant. We suggest that Wg signals the coordinated development of pre- and postsynaptic compartments.

# Marzo et al 2016 Curr Biol “Reversal of Synapse Degeneration by Restoring Wnt Signaling in the Adult Hippocampus“ <https://www.ncbi.nlm.nih.gov/pmc/articles/PMC5070786/>

# “Synapse degeneration occurs early in neurodegenerative diseases and correlates strongly with cognitive decline in Alzheimer’s disease (AD). The molecular mechanisms that trigger synapse vulnerability and those that promote synapse regeneration after substantial synaptic failure remain poorly understood. Increasing evidence suggests a link between a deficiency in Wnt signaling and AD”

# Elliott et al 2018 “A role for APP in Wnt signalling links synapse loss with β-amyloid production <https://www.nature.com/articles/s41398-018-0231-6>

# “In Alzheimer’s disease (AD), the canonical Wnt inhibitor Dickkopf-1 (Dkk1) is induced by β-amyloid (Aβ) and shifts the balance from canonical towards non-canonical Wnt signalling. Canonical (Wnt-β-catenin) signalling promotes synapse stability, while non-canonical (Wnt-PCP) signalling favours synapse retraction; thus Aβ-driven synapse loss is mediated by Dkk1. Here we show that the Amyloid Precursor Protein (APP) co-activates both arms of Wnt signalling through physical interactions with Wnt co-receptors LRP6 and Vangl2, to bi-directionally modulate synapse stability.”

From this review: Libro et al 2016

<https://www.sciencedirect.com/science/article/pii/S0024320516303800?via%3Dihub>

“Overall, all the studies reported here, and summarized in [Table 1](https://www.sciencedirect.com/science/article/pii/S0024320516303800?via%3Dihub#t0005), have shown that Aβ promotes GSK-3β activity by stimulating the expression of DKK1. In parallel, GSK-3β promoted the inactivation of the Wnt canonical pathway and NFTs formation by phosphorylating β-catenin and Tau. In addition, GSK-3β can further exacerbate AD pathology by triggering inflammatory and apoptotic pathways ([Fig. 2](https://www.sciencedirect.com/science/article/pii/S0024320516303800?via%3Dihub#f0010)). Altogether, these findings suggested that that loss of function of the Wnt canonical pathway is implicated in AD pathogenesis, while the activation of the Wnt canonical pathway attenuates AD pathology by protecting neurons against Aβ-toxicity, by reducing synapse loss and ameliorating cognitive performances.”

There is a special issue about this topic:

This is from the introduction article of the issue:

<https://academic.oup.com/jmcb/article/6/1/1/875775>

“Deregulation of Wnt signaling has been proposed as a key contributor to the pathogenesis of neurodegenerative disorders including Alzheimer's disease (AD) and Parkinson's disease (PD). This increasing knowledge of the specific roles of Wnt signaling cascades during different stages of life has suggested innovative therapeutic strategies for the treatment of neurodegenerative diseases. The reviews under the theme ‘Wnt Signaling Cascades in Neurodevelopment, Neurodegeneration and Regeneration’ published in this issue of JMCB”

4)"This study integrates for the first time the oncogenic nature of glioma with the neuronal degeneration caused by Wg depletion" this sentence is not appropriate, see above.”

We have modified the sentence as follows*: “This study integrates for the first time the oncogenic nature of glioma with the neuronal degeneration caused by TMs expansion.”*
Minor: the vampire picture in the model is not appropriate for this journal. You migth find a better way to represent your "vampirization" hypothesis.

We have modified the figure accordingly.

Rev. 2:
I have not been able to read the whole paper. It is formally very disorganized and contain multiple inconsistencies.

We have addressed all the concerns raised by the reviewer.
Below, some examples:
A crucial part, is the list of references. I have tried to read some of the references but is impossible to access to them. The list of references is totally inconsistent with the refs indicated in the text. If I can't read the papers cited, I can't evaluate the work.
We apologize for this error; some problems with the Endnote drove us to disorganize the references. We have corrected all of them and the new ones properly.
A couple of examples:
1- The authors write: WNT signaling has long been suggested as a hallmark in gliomagenesis associated with the proliferation of stem-like cells in human GBs [48]. The reference 48 in the references sections corresponds to “Cytoneme-mediated contact-dependent transport of the Drosophila decapentaplegic signaling protein.” Roy S, Huang H, Liu S, Kornberg TB. (PMID: PMID: 24385607), which has nothing to do with WNT signaling and gliomagenesis.

We have corrected this reference : Kahlert UD, Suwala AK, Koch K, Natsumeda M, Orr BA, et al. (2015) Pharmacologic Wnt Inhibition Reduces Proliferation, Survival, and Clonogenicity of Glioblastoma Cells. J Neuropathol Exp Neurol 74: 889-900.

2- The mechanisms of Wg delivery are currently under debate. This protein was initially described as a secreted protein [61]. Recent studies have proved that Wg secretion is not necessary for Drosophila development [62].

We have fixed this and referenced to the correct papers:

Bejsovec A, Martinez Arias A (1991) Roles of wingless in patterning the larval epidermis of Drosophila. Development 113: 471-485.

Alexandre C, Baena-Lopez A, Vincent JP (2014) Patterning and growth control by membrane-tethered Wingless. Nature 505: 180-185.

In the references part:
61. Scherer HJ. A Critical Review: The Pathology of Cerebral Gliomas. J Neurol Psychiatry. 1940;3(2):147-77. Epub 1940/04/01. PubMed PMID: 21610973; PubMed Central PMCID: PMC1088179.
62. Boyle M, Bonini N, DiNardo S. Expression and function of clift in the development of somatic gonadal precursors within the Drosophila mesoderm. Development. 1997;124(5):971- 82. Epub 1997/03/01. PubMed PMID: 9056773.
We have fixed this
“To determine if TM-like processes expand as a consequence of the increase in the number of GB cells, we quantified the volume of the TM-like processes and divided by the number of glial cells (Figure 1R). The results show that TM-like processes volume/glial cell number ratio is higher in GB and therefore we conclude that TM-like processes expand in GB.”
The authors aimed to determine whether “TM-like processes expand as a consequence of the increase in the number of GB cells”. At the end of the paragraph they conclude, “we conclude that TM-like processes expand in GB”. But this does not answer the question they opened in the beginning of the paragraph: “if TM-like processes expand as a consequence of the increase in the number of GB cells” It is therefore very difficult to follow and understand.
We have clarified this section as follows: *“The results show that TM-like processes volume with respect to the glial cell number ratio is higher in GB, and therefore we conclude that TM-like processes expand in GB beyond the increase in the number of glial cells”.*
Fig S1. A, B, show panels labeled at the top if the Fig as
• LifeactinGFP (green) and the panels below show black and white images
• Repo-Ihog-RFP (red) and the panels below show black and white images
• DAPI (blue) and the panels below show merged image.
Why? Again, this inconsistencies in the format make the manuscript very difficult to follow. The Figures should be self-explanatory and easy to digest, and this is not the case in this work.
We apologize for this mislabelling, we have corrected this in all the figures
In the text: “Moreover, using methods that were used to prevent epithelial cytoneme formation (by downregulating neuroglian (nrg-RNAi)) [46], we found that the TM-like processes in GB were also reduced (Figure S1 H-I´´).” Those panels show two examples of Glioma; NrgRNAi (same genotype in both of them). The authors claim TM are reduced. Reduced when compared to what? Where is the control to compare with? If they claim they are reduced, this should be quantified. The authors should show these data in a more rigorous manner and provide always controls that allow to, at glance, compare with the experimental conditions. Without this, the conclusions are not supported by the results shown.
We have included the control and quantifications of network volume (FigS1 H-J).

The writing and grammar is, as in the previous version, very poor. See, eg: Ihog (Interference hedgehog), which is a type 1 membrane protein shown to mediate the response to the active Hedgehog (Hh) protein signal [42], which accumulates in the epithelial cytonemes [43,44].
We have corrected the writing and grammar and it has been revised by a english native speaker.

As the previous version, this manuscript is below the basic standars required for publication in Plos Biol.

We have addressed all the concerns raised by the reviewers, therefore we hope that this improved version of both manuscripts fused is now acceptable for publication in PLoS Biol.

Rev. 3:
The revised manuscript “Glioblastoma cells vampirize WNT from neurons and trigger a JNK/MMP signaling loop that enhances glioblastoma progression and neurodegeneration” has now combined two previous manuscripts and re-organized the story, making it more comprehensive. The paper has been improved.
Most of the concerns from the last review have been addressed with proper experiments completed, except for the question #6. I could not agree that unmarked remaining non-glial cells are neurons. There are mixed cell types in tumor microenvironment including endothelial cells, fibroblasts and immune cells. In addition, as the authors demonstrated, neurons are subjected to degeneration and apoptosis. How would they assume that neurons largely existed and were all myr-RFP/Ihog-RFP negative cells? Neuron markers are strongly recommended to show colocalization with Wg reporters.

Myr-RFP/Ihog-RFP markers are expressed under the control of the specific enhancer “Repo-Gal4”. This driver is restricted to glial lineage as we showed in 2015 (<https://www.ncbi.nlm.nih.gov/pubmed/28405401>). Therefore, only glial cells are marked with RFP.

According to the previous literature, there are no evidences of fibroblasts, endothelial cells or immune cells in the *Drosophila* brain tumor microenvironment.

In the Drosophila nervous system, the blood-brain barrier is formed by perineurial glial cells and subperineurial glial cells form septate junctions (<https://www.ncbi.nlm.nih.gov/pmc/articles/PMC4231875/>). Both types of cells are also Repo+.

In *Drosophila*, neuroectodermally derived cells carry out many immune functions in the CNS, suggesting that a single cell type carries out the role of macro- and microglia. While these cells are generally called glia and all express the pan-glial marker Repo, different subtypes seem to accomplish specific functions (<https://www.nature.com/articles/s42003-018-0070-2>). The cortex glia act as non-professional phagocytes clearing up apoptotic cells in the developing nervous system (<https://onlinelibrary.wiley.com/doi/abs/10.1002/glia.21098>).

Confocal images of brains stained for neuronal markers (ELAV and HRP) in Figure 1F-G show that the cells next to glial cells, are neurons. In the case of the glioma, we have also detected ELAV or HRP positive cells inside of the glioma nests.
6. In Fig 4E-H and Fig S7B-G, Wg pathway reporters were only co-stained with glia but not with neuron. How was the conclusion drawn that the reporter activities were down in neurons?
In both figures, control samples (without GB) are included and arrows indicate the activity of Wg reporters in neurons (Figure 3-E, G and Figure S5 B, D and F). Please take into consideration that in the Drosophila model, all glial cells in control brain and glioma were marked with myr-RFP/Ihog-RFP, so the unmarked remaining cells should be neurons.

Yes, this is correct, glial cells are marked with RFP in confocal images and, ELAV or HRP staining show that among the neighbouring cells to the glioma, there are neurons. In addition, electron microscopy images show glial cells marked with HRP (RepoGal4>UAS-HRP), the cells next to HRP+ glial cells show neuronal morphology (Figure 1H-N).

<https://www.jneurosci.org/content/36/11/3170> See Figure 9B-C

<https://journals.plos.org/plosone/article?id=10.1371/journal.pone.0131250>

See Figure 1C, 3B, 3D

We cannot discard that other cell types are included in these nests and also silence Wg pathway, and this has been taken into consideration in the discussion of the results. However, our main interest is to study the neurodegeneration associated to the progression of the glioma and the mechanisms that mediate this degeneration.

Overall, the quality of the revised manuscript is improved, and a further revised version is in favor of publication.

We would like to thank the reviewer for his/her comments

Thank you in advance for your help.

With best regards,

Sergio [scasas@cajal.csic.es](mailto:scasas@cajal.csic.es)

Madrid, September 2019
